# Gene association analysis of an osteopontin polymorphism and ketosis resistance in dairy cattle

**DOI:** 10.1038/s41598-023-48771-5

**Published:** 2023-12-06

**Authors:** Edyta A. Bauer, Dominika Kułaj, Sebastian Sawicki, Joanna Pokorska

**Affiliations:** Department of Animal Reproduction, Anatomy and Genomics, Faculty of Animal Science, University of Agriculture in Krakow, Al. Mickiewicza 24/28, 30-059 Krakow, Poland

**Keywords:** Genetics, Immunology, Molecular biology

## Abstract

The aim of this study was to identify the c.495C > T polymorphism within exon 1 of the osteopontin gene (OPN), and to analyze its association with susceptibility to ketosis in Polish Holstein–Friesian (HF) cows. The study utilized blood samples from 977 HF cows, for the determination of β-hydroxybutyric acid (BHB) and for DNA isolation. The c.495C > T polymorphism of the bovine osteopontin gene was determined by PCR–RFLP. The CT genotype (0.50) was deemed the most common, while TT (0.08) was the rarest genotype. Cows with ketosis most often had the CC genotype, while cows with the TT genotype had the lowest incidence of ketosis. To confirm the relationship between the genotype and ketosis in cows, a weight of evidence (WoE) was generated. A very strong effect of the TT genotype on resistance to ketosis was demonstrated. The distribution of the ROC curve shows that the probability of resistance to ketosis is > 75% if cows have the TT genotype of the OPN gene (cutoff value is 0.758). Results suggest that TT genotype at the c.495C > T locus of the OPN gene might be effective way to detect the cows with risk of ketosis.

## Introduction

Ketosis, one of the metabolic diseases found in dairy cattle, is characterized by increased levels of ketone bodies (β-hydroxybutyric acid (BHB), acetylacetic acid, and acetone) in body fluids. Ketone bodies are formed by ketogenesis and result from the oxidation of free fatty acids in the liver. Whitaker et al.^1^ indicate that in healthy cows, BHB levels range from 0.6 to 1.0 mmol/L. There are two classification schemes in the literature for ketosis. The first, which divides ketosis into subclinical (SCK) and clinical (CK), is based on the measurement of BHB levels in blood, milk or urine. In general, SCK refers to cows with blood BHB levels ranging from 1.2 to 1.4 mmol/L^2^, although other studies have suggested blood BHB levels indicative of SCK to be from 1.0 mmol/L^3^, 1.2 mmol/L^2,4^ or 1.4 mmol/L^3,5^. In addition to significant hyperketonemia (blood BHB levels >3 mmol/L^5^), CK is characterized by hypoglycemia and symptoms such as loss of appetite, weight loss, and decreased milk yield^6^. The second classification scheme divides ketosis into three types: I, II, and III. Type I and Type II ketosis are analogous to Type 1 and Type 2 diabetes in humans^6,7^. Type I ketosis affects cows between the third and sixth weeks of lactation, in which the demand for energy (including glucose) due to increasing milk production is so high that it exceeds the body's ability to meet its needs from feed^7^. Satisfying the body's energy needs is accomplished by increasing the secretion of ketone bodies, and hyperketonemia in this case is accompanied by very low levels of insulin and plasma glucose^6^. Type II ketosis occurs immediately after parturition and is associated with hepatic steatosis. Cows with Type II ketosis have high concentrations of both insulin and blood glucose at the time of diagnosis of hyperketonemia^6^. Type III ketosis is attributable to the consumption of feed rich in ketogenic precursors (including butyric acid), and low energy or high protein content in the daily ration, as well as poor quality silage. The onset of Type I and Type II ketosis can be followed by the onset of conditions including rumen acidosis and displacement of the digestive tract^8^. The occurrence of both CK and SCK severely affects the economics of production on dairy farms, due to their impact on the milk yield, the incidence of other diseases, and the costs of veterinary treatment and loss of cows from the herd due to premature death^6,9^. Moreover, it has been shown that cows with SCK often produce milk with higher somatic cell counts^10^. It is extremely important for breeders to diagnose SCK quickly and cost-effectively, since, according to some estimates, the prevalence of undiagnosed cases of this disease in dairy herds is in the range of 15–40%^6^. Measuring serum or milk BHB levels continues to be widely used for the diagnosis of ketosis in cows^9^. Recently, a method to diagnose subclinical ketosis based on the analysis of milk samples using Fourier Transform Infrared Spectroscopy to determine the content of such parameters as BHB (mmol/L) or acetone (mmol/L) has been proposed^11^. Vlček et al.^12^, on the other hand, suggested that the evaluation of other milk parameters could be a good indicator for predicting ketosis in cows, and showed that the percentage fat/protein ratio in cows' milk can be considered a non-invasive and low-cost method to screen for metabolic disorders. The use of in silico testing to develop a panel of putative markers like SNPs (Single Nucleotide Polymorphisms) for diagnosing genetic resistance of cows to ketosis is another possible approach. Certain genes encoding proteins involved in a range of metabolic processes, including the synthesis and degradation of fatty acids and ketone bodies, gluconeogenesis, lipid mobilization and the citric acid cycle, have been shown to contain SNP-type polymorphisms that may be associated with resistance to the disease^13^. Moreover, genome-wide association studies have identified candidate genes for resistance or susceptibility to ketosis in Holstein cattle^14^ and putative markers for disease resistance in the Jersey breed^15^. Being a metabolic disease, ketosis has a low heritability, ranging from 0.04 to 0.14^16^, but it has been suggested that resistance could be improved through genetic selection^15^. Osteopontin (OPN) is a glycoprotein with versatile properties that is expressed in many cell types (osteoclasts, osteoblasts, teeth, epithelial cells of the breast, kidney, skin, nerve cells, vascular endothelial cells, T lymphocytes, macrophages, NK cells, and dendritic cells). In addition to its primary function in the skeletal system, OPN plays an important role in the operation of the animal immune system as demonstrated by its ability to induce chemotaxis and migration of immune cells to the site of inflammation, and participation in processes such as angiogenesis, cell adhesion, apoptosis, regulation of inflammation, and wound healing. In addition, it acts as an opsonin and, by attaching to bacterial variants, enhances phagocytosis^17^. Osteopontin is also considered to be a putative biomarker of metabolic changes occurring in dairy cows^18^. The purpose of this study was to examine the polymorphism at the c.495C > T *locus* (rs109659827) of the osteopontin gene and its association with SCK susceptibility in Polish Holstein-Friesian cows of the Black and White breed.

## Results

**Figure 1:**
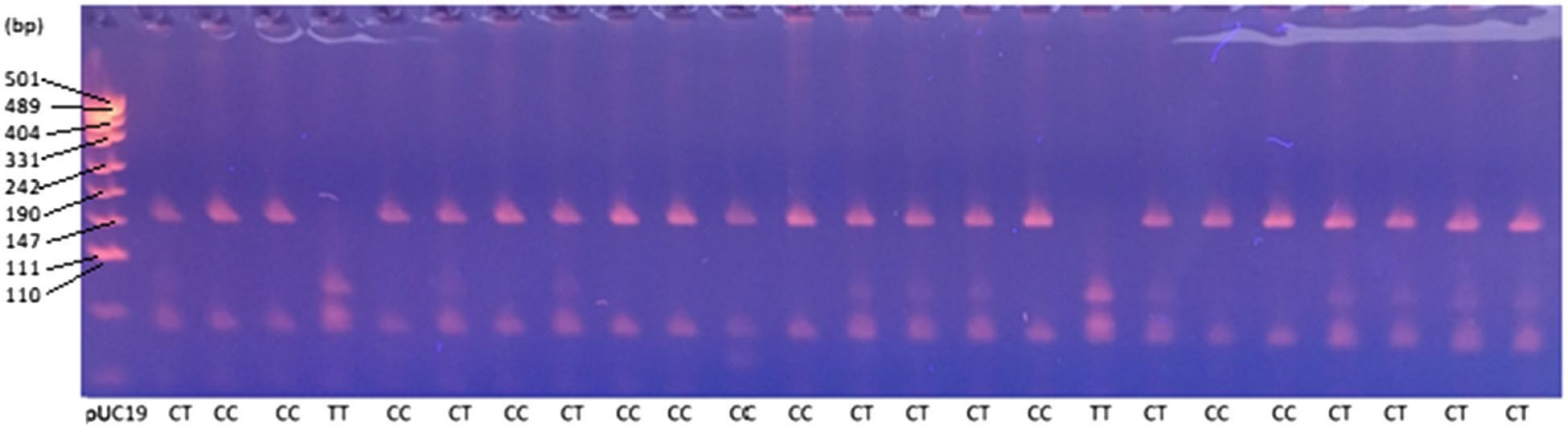
Electrophoretic separation of the PCR–RFLP within *locus* c.495C > T osteopontin gene.

Genotypes within the analyzed *locus* c.495c > t (RS109659827) of the osteopontin gene were determined on the basis of the electrophoretic separation of the RFLP sections (RFLP bands) in agarose gel (Fig. 1).

The substitution of cytosine by thymine is a missense mutation type, which means that it causes an amino acid substitution in the protein coded by this gene (proline by leucine). The presence of two alleles in the analyzed locus: C and T and three genotypes: CC, CT and TT were observed in the cow population. Allele C appeared with a frequency of 0.71, and allele T with a frequency of 0.29. The most common genotype was CT, which was identified in 50% of the animals. The frequencies of the other genotypes were 0.42 for the CC genotype and 0.08 for TT, respectively. Table 2 presents the distribution of genotypes (CC, CT, TT) within the examined *locus* of the osteopontin gene (n = 977).

In the subsequent step, the power of the test (ability of bivariate arrays to reject the hypothesis) with regard to the relationship between the traits ketosis 0/1 × OPN (exon 1) was determined, and bivariate arrays (w x k) were generated via the bar method. The power of the selected test was estimated based on the sample size^22^. Table 2 shows the genotype abundance (CC, CT, TT) for the *locus* analyzed, in relation to the presence of ketosis. The bivariate (2 × 3) table (Table 1) of the relationship between the specific feature. contains a sufficient number of occurrences and shows a strong correlation between traits.

**Table 1:** 2 × 3 contingency table showing the abundance of CT, CC and TT alleles for OPN (exon1) with the occurrence of ketosis 0 or 1.

Ketosis 1: 0	OPN (exon 1)			
	CT	CC	TT	Total
0	462	385	81	928
1	23	25	1	49
Total	485	410	82	977

**Figure 2:**
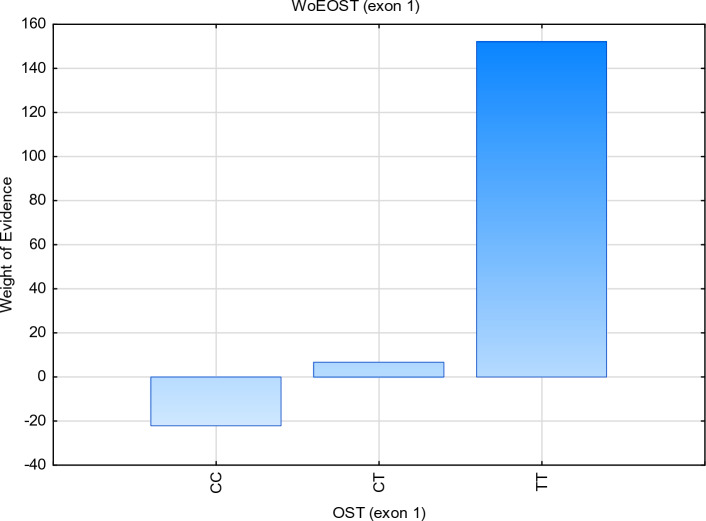
Weight of Evidence (WOE) for osteopontin genotype (CT, CC and TT) with subclinical ketosis.

**Figure 3:**
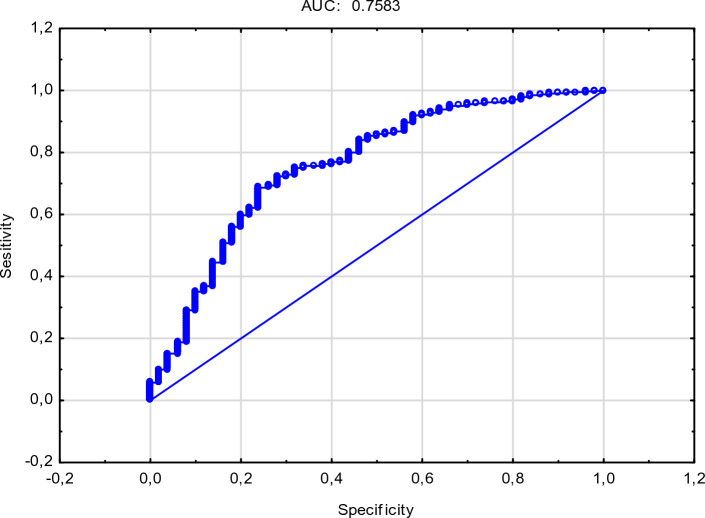
Response operating characteristic curve (ROC) of the OPN for TT genotype.

In order to confirm the relationship between the studied genotypes (CC, CT, TT) at the c.495C > T *locus* of the OPN gene, a weight of evidence (WoE) was generated to objectively illustrate the interaction of spatial data. By using the graph of *WoE* values, CC, CT, TT genotypes were combined, and interval boundaries were generated automatically according to the distribution of the variable (ketosis 0:1). Figure 2 shows *WoE* values for ketosis and genotypes CC, CT, and TT. Higher *WoE* values indicate a higher probability of absence of ketosis for the TT genotype, at the c.495C > T *locus* of the OPN gene, while similar *WoE* ranges for the CC and CT variables implies a similar high risk of ketosis. Such categories can be combined into a common value for possible ketosis. Figure 2 presents a graph of *WoE* values for TT genotypes, one of the potential factors identifying ketosis-resistant cows. This graph illustrates positive and negative values of the weight of evidence in successive frequency subgroups. The regularity shown in the graph (Fig. 2) points to a strong relationship between the examined trait (TT) and the dependent variable (ketosis). The distribution of the ROC curve for the TT genotype of the OPN gene shows the ability to detect healthy individuals (Fig. 3). The cutoff value separating normal results from those considered abnormal is 0.758.

## Discussion

A number of authors have worked on the development of a simple and inexpensive method for the early detection of ketosis in cows^3,19^. These studies mainly focused on changes in milk composition in cows with ketosis, and more specifically on changes in the level of ketone bodies in milk such as BHB and acetone. Using different approaches, several authors have found similar cutoff values for the circulating levels of BHB. With a cutoff ≥1.2 mmol BHB/L, Bauer and Jagusiak^19^ reported an area under the ROC curve (AUC) value of 0.861, while Chuang et al.^23^, using fibroblast growth factor-FGF:21 as a biomarker for ketosis in cows, reported an AUC of 0.95. In their investigation of Type I and Type II ketosis, Delić et al.^7^ reported an AUC of 0.74 using a cutoff of >1.2mmol BHB/L.

The bovine osteopontin gene is located on chromosome 6 and consists of 7-9 exons, depending on the transcript. Many polymorphic sites have been located in both coding and non-coding parts of this gene. Some of these sites have been analyzed in terms of the relationship between specific genotypes and milk production and chemistry^24,25^ and the occurrence of diseases such as mastitis, postpartum ovarian cysts, and abscess^25^. Much of the research on OPN gene polymorphism in cattle has focused on the c.8514 C>T site located in intron 4^25,26^. To our knowledge, this is the first study to investigate the relationship between polymorphisms at the c.495C>T *locus* (rs109659827) of the OPN gene and dairy traits and diseases in cattle, and the first to analyze the relationship between polymorphisms in the OPN gene and ketosis in this species. It has been suggested that osteopontin could be a biomarker of the metabolic changes occurring in dairy cows with ketosis, as it was shown to be one of 11 proteins with decreased urinary levels in affected cows, suggesting that OPN plays an important role in the inflammatory process during this disease^18^. Polymorphisms in the promoter region of the OPN gene have been shown to affect OPN levels in milk during late lactation, and although the exact role of OPN remains to be clarified, it has been suggested that it may be involved in tissue remodeling and thus in reducing the incidence of infection during this period^27^. The transition period includes not only metabolic, but also immunological, changes and is accompanied by an increased risk of infection, as demonstrated by an almost four times higher incidence of intramammary infections during the drying-out period than during the lactation period^28^. A study which measured pro-inflammatory cytokines in three groups of cows: before, during, and after ketosis, demonstrated that cows before ketosis and in ketosis showed significantly higher serum levels of interleukin-6 (IL-6) and tumor necrosis factor (TNF) compared to healthy individuals, suggesting that the cows experienced chronic low-grade inflammation even before ketosis^29^. It has been speculated that the source of systemic inflammation in cows in the transition period may be a subclinically infected mammary gland -, but this conjecture requires further investigation^6^. The release of ketone bodies during the transition period may be a cow's response to reduce the risk of systemic inflammation systemic inflammation. A study by Youm et al.^30^ showed that BHB directly inhibits the assembly and activation of the NLRP3 inflammasome, which is a key regulator of the innate immune system and is involved in inflammation and obesity-related diseases in humans^31^. The NLRP3 inflammasome is a functional complex activated by molecular patterns associated with cellular damage and pathogens, responsible for the production of pro-inflammatory cytokines such as interleukins: 1β (IL-1β) and 18 (IL-18). However, the activity of the NLRP3 inflammasome also includes maintaining intestinal mucosal homeostasis and controlling the composition of the intestinal microbiome, so it may be an important protective factor in metabolic diseases^32^. A study of the effect of recombinant osteopontin administration on protection against cerebral infarction in rats showed that OPN inhibited the activation of the NLRP3 inflammasome^33^, suggesting that it may also be involved in inhibiting systemic inflammation during ketosis.

## Material and methods

### Methods

#### Approval for animal experiments

The prof. Dorota Ziyba-Przybylska as a Chairman of ethics review board and Dean of the Faculty of Animal Science, University Agriculture in Krakow considers that this type of project does not fall under the legislation for the protection of animals used for scientific purposes. The animals used in this manuscript were not slaughtered and no special treatment was applied. The authors obtained data from databases belonging to breeders' association. Blood samples were collected by technicians employed by farms or the breeders' association as part of standard veterinary procedures.

### Initial dataset

The original dataset consisted of 979 unique test-day (TD) records for Polish Holstein–Friesian cows, provided by the Polish Federation of Cattle Breeders and Dairy Farmers. Heifers were kept in three different barns located in the Lower Silesia province (Poland). The animals were kept in free stall barns and fed a Total Mix Ration. The records were from the first eight lactations, grouped into six categories of lactation (1, 2, 3, 4, 5, ≥6), and included cows calved in 2020 and 2021. The data included milk traits like milk yield, fat, protein, lactose and dry matter percentages, milk urea concentration, somatic cell count (SCC) and BHB. All milk variables were recorded as continuous traits and were not assigned to categories. Table 2 shows the descriptive statistics of the initial dataset. Some records (n=2) were excluded from further analysis (ketosis test) due to contamination.

**Table 2:** Number of animals tested (n = 979), mean and standard deviation of milk variables and Β- hydroxybutyrate concentration (BHB) in blood according to lactation number.

Item	Lactation 1	Lactation 2	Lactation 3	Lactation 4	Lactation 5	Lactation ≥ 6
N	326	291	197	87	50	28
BHB	0.47 ± 0.24	0.44 ± 0.21	0.47 ± 0.35	0.43 ± 0.28	13.22 ± 92.87	0.47 ± 0.30
Milk variables						
Milk	24.84 ± 9.53	27.96 ± 13.12	27.77 ± 14.13	24.65 ± 12.02	23.71 ± 12.85	20.18 ± 8.93
Fat	4.08 ± 0.86	4.17 ± 0.89	4.02 ± 0.82	3.97 ± 0.77	4.03 ± 0.78	4.03 ± 0.69
Protein	3.60 ± 0.35	3.57 ± 0.36	3.49 ± 0.35	3.43 ± 0.36	3.43 ± 0.37	3.46 ± 0.33
Lactose	4.91 ± 0.19	4.82 ± 0.58	4.74 ± 0.22	4.72 ± 0.23	4.66 ± 0.24	4.67 ± 0.29
Dry matter	13.33 ± 1.53	17.64 ± 76.81	12.99 ± 1.35	12.93 ± 0.99	12.65 ± 2.04	12.62 ± 2.34
Urea	235.82 ± 66.04	251.29 ± 101.93	250.27 ± 55.00	252.49 ± 61.73	254.06 ± 55.66	250.53 ± 67.09
SCC	172.46 ± 394.66	228.40 ± 473.98	344.50 ± 778.30	376.34 ± 919.72	479.15 ± 829.54	825.31 ± 2425.46

N Number of cows, mean and standard deviation of blood β-hydroxybutyrate concentration (BHB)(mmol/L), milk yield(kg), fat(%), protein(%), lactose(%), urea concentration(mg/L), somatic cell score (SCC)(1000/mL) and dry matter of milk(%).

### Laboratory analysis

Blood samples were collected from the tail vein (n=979), using disposable evacuated blood collection tubes containing an anticoagulant, potassium EDTA. Blood samples were used for DNA isolation and testing of β-hydroxybutyrate acid (BHB) content. Genomic DNA was isolated using the MasterPure™ DNA Purif Kit (Lucigen). The quantitative and qualitative analysis of the isolated DNA was verified spectrophotometrically using a Nano Drop 2000 analyser (Thermo Scientific, USA). The isolated DNA was stored at − 25 °C prior to further analysis. Genotypes within the analyzed *locus* c.495C > T (rs109659827) of exon 1 were determined using the Sanger sequencing method and the PCR-RFLP reaction. The F- 5’ GTGTGTGCCTGTGTTTGTTC 3’ and R- 5’ GAGAAGAGTCCAGTCCCCTG 3’ sequences were used to flanking exon 1 region of the osteopontin gene. These primers were designed using the Primer3Plus software based on the reference sequence (GenBank Accession No. AC_000163.1) and are patent pending under Polish law (Bauer, E.A., Pokorska, J., Kułaj, D., Borówka, W., Zestaw starterów i jego zastosowanie do wykrywania genetycznej odporności krów na ketozę oraz sposób wykrywania genetycznej odporności na ketozę u krów. 2022. P.441536 (WIPO ST 10/C PL441536) University of Agriculture in Krakow). PCR reaction was conducted in the C1000 Thermal Cycler (Bio-Rad) in 0.2 uL tubes, which included the following reagents: 1x Taq buffer + KCl—MgCl_2_ (Thermo Scientific, USA), 0.175 µM of each primer, 1.125 mM MgCl_2_, 0.105 mM of each dNTP, 1.75 units Taq polymerase (Thermo Scientific, USA), 1% DMSO and 100–150 ng template DNA. The PCR thermal program contained the following steps: initial denaturation—95°C for 5 min, cycling—95 °C for 40 s, 65 °C for 45 s, 72 °C for 45 s (repeated in 34 cycles), and final elongation—72 °C for 5 min. Next, PCR products were incubated with MnlI restriction enzyme (10 U/μL, BioLabs, UK) in 37 °C for 3h. The composition of the RFLP reaction mixture was as follows: 3.6 µL ultra-pure water, 1 μL 1x buffer (BioLabs, UK), 0.4 μL MnlI endonuclease and 5 uL PCR products. PCR-RFLP bands were separated in 3.5 % agarose gel (Merck, German) with ethidium bromide (0.5 µg/mL, Merck, German). Electrophoresis was performed at 100 V for 90 min in the presence of the pUC19 DNA/MspI marker using the Mini-PROTEAN device (Bio-Rad) (Thermo Scientific, USA).

The β-hydroxybutyrate acid concentrations in blood were measured using a spectrophotometer (model: UV-5100, Shandong, China). For further analysis, the BHB thresholds were used as the diagnostic reference for subclinical ketosis ≥1.0 mmol/L^3,19^. Cows with circulating BHB lower than the pre-defined threshold were considered to be healthy. A total of 977 cows had a blood BHB level in this range. The input variable was defined as the cow’s health status, i.e., a zero-one parameter: healthy cow = 0 and ketosis-affected cow = 1. Table 3 shows the record of osteopontin genotype on cows with subclinical ketosis.

**Table 3:** Records of osteopontin genotype (CC, CT, TT) on cows with subclinical ketosis (n = 977).

N	Genotype					
	CT	k	CC	k	TT	k
977	485	23*	410	25	82	1*

Number of cows with subclinical ketosis (k) according to three genotypes (CT, CC, TT).

*Two samples were contaminated (ketosis test).

### Statistical analysis

The frequencies of genes and genotypes at the c.495C > T (rs109659827) osteopontin *locus* were determined by a direct count. To identify and understand the prediction of quantitative analysis of evidence factors and the links type, the Weight of Evidence (*WoE*) was chosen as an effective tool. The method is objective, especially in the choice of weighting factors. The *WoE* in this context was considered symbolic and used as a summary interpretation of the evidence. The model describes and analyzes the interaction of spatial data, and provides intellectual support for the policy makers, which might be an effective tool^20^. The *WoE* a categorical predictor is commonly defined as follows:

$$WoE=\left[In\left(\frac{Relative \,Frequency\, of \,Goods}{Relative\, Frequency \,of \,Bad}\right)\right]*100$$

The next stage was to create a visual representation of the possible relationships between the two sets of categorical data. The three-way table is concerned with the investigation of the simultaneous effects of two nominal variables, say A, B and C, called factors (CC, CT, TT genotypes of osteopontin gene). Each combination of a factor level of A and a factor level of B is a treatment (ketosis 1:0). These factors can take different values known as levels. The categories are labelled at the top and left side of the table, with the frequency (count) information appearing in the four (or more) interior cells of the table. The “totals” of each row appear at the right, and the “totals” of each column appear at the bottom. The sum of row totals equals the sum of the column totals (Fig. 2).

In the final stage of the preliminary process, sensitivity and specificity were calculated within a receiver operating characteristic (ROC) curve^21^. ROC curves were produced to provide a graphic illustration (Fig. 3) of the accuracy of the TT genotype and ketosis (1 or 0). In the current study, Sensitivity was defined as the proportion of subclinical ketosis cows (TT genotype) that tested negative.

Specificity was defined as the proportion of subclinical ketosis cows (TT genotype) that tested positive. An optimum cut-off point is a point located on a ROC curve that is close to the point having coordinates (0.1). Sensitivity, and specificity were calculated as indicated by Sonego et al.^21^.

### Institutional review board statement

All animals used in this study were kept according to the Polish legislation for dairy cows production. The collection of samples and care of the animals used in this study followed the guidelines for experimental animals established by the Animal Care Committee and were ap-proved by the Ethics Committee of University of Agricultural in Krakow, Poland.

## Conclusion

Our results demonstrate that genotyping at the c.495C > T (rs109659827) locus of the OPN gene using our PCR–RFLP assay supports the identification of ketosis-resistant animals, providing an effective, quick, and inexpensive early screening tool to reduce the incidence of ketosis in dairy herds. Our PCR Proposal—RFLP Assay is a quick and inexpensive early screening tool for predicting resistance to ketosis. This work is an important contribution to research to find SNPs that could be used in marker-assisted selection in cattle. The research should be continued and include the analysis of other polymorphic sites that may be located within the OPN gene (Supplementary Information).

### Supplementary Information

Supplementary Information.

## Data Availability

None of data were deposited in an official repository. Data may be available upon request by contacting the corresponding author. Corresponding author E.A. Bauer, email: e.bauer@urk.edu.pl.
