# Supplementary material for: Gene association analysis of an osteopontin polymorphism and ketosis resistance in dairy cattle
Source: Sci Rep. 2023 Dec 6;13:21539. doi: 10.1038/s41598-023-48771-5 (PMC10700331; doi:10.1038/s41598-023-48771-5)
Supplement: Supplementary file 1 — Supplementary Information. [file 41598_2023_48771_MOESM1_ESM.pdf]

Gene association analysis of an osteopontin polymorphism and ketosis resistance in dairy cattle

Edyta. A. Bauer, Dominika Kułaj, Sebastian Sawicki, Joanna Pokorska

Department of Animal Reproduction, Anatomy and Genomics,
Faculty of Animal Science,
University of Agriculture in Krakow,
Al. Mickiewicza 24/28, 30-059 Krakow, Poland
Correspondence should be addressed to e-mail: e.bauer@urk.edu.pl

Laboratory Analysis

Electrophoretic separation of the PCR-RFLP within locus c.495C > T osteopontin gene

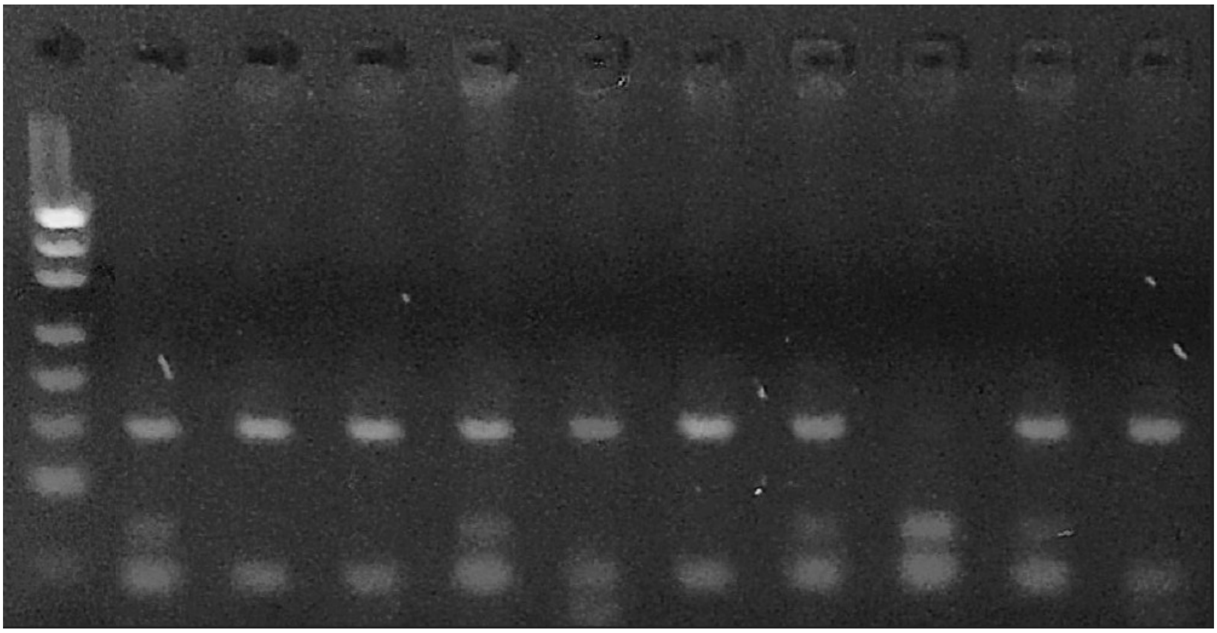

Image 300 DPI

The following banding patterns were obtained to notice the genotypes of the animals:

OPN - CC:	147 pz		57 pz	30 pz
OPN - CT:	147 pz	82 pz	65,57 pz	30 pz
OPN - TT:		82 pz	65,57 pz	30 pz

The osteopontin genotype (CT, CC, TT) for exon 1 and BHB for all cattle (n=979)

No.	BHB0/1	OPN(exon 1)
1	0	CT
2	0	CT
3	0	CT
4	1	CC
5	0	CT
6	0	TT
7	0	CT
8	0	CT
9	0	CC
10	0	CT
11	0	CT
12	1	CT
13	0	CC
14	0	TT
15	0	CT
16	0	CT
17	0	CT
18	0	CC
19	0	CC
20	1	CT
21	0	CT
22	0	CC
23	0	CC
24	0	CC
25	0	CT
26	0	CT
27	0	TT
29	0	CC
30	0	CT
31	0	CT
32	0	CT
33	0	CC
34	0	CC
35	0	CC
36	0	CC
37	0	CT
38	0	CC
39	0	CT
40	0	CT
41	0	CC
42	0	CT
43	1	CC
44	0	CT
45	0	CT
46	0	CT

48	0	CT
49	0	CC
50	0	CC
51	0	CC
52	0	CT
53	0	CT
54	0	CC
55	0	CT
56	0	CT
57	1	CT
58	0	CT
59	0	CC
60	0	CT
61	0	CT
62	0	TT
63	0	CT
64	0	CC
65	0	CC
66	0	CT
67	0	CC
68	0	CT
69	0	CT
70	0	CC
71	0	CC
72	0	CT
73	0	CT
74	0	CC
75	0	CC
76	0	CT
77	0	CT
78	0	CT
79	0	CC
80	0	CT
81	0	CC
82	0	CT
83	0	CC
84	0	CT
85	0	CC
86	0	CC
87	0	CC
88	0	TT
89	0	CC
90	0	CC
91	0	CT
92	0	CC
93	0	CC

94	0	CC
95	0	CT
96	0	CC
97	0	CC
98	0	CC
99	0	CT
100	0	CT
101	0	CC
102	0	TT
103	0	CT
104	0	CC
105	0	CC
106	1	CT
107	0	CT
108	0	CT
109	0	CT
110	0	CC
111	0	CC
112	0	TT
113	0	CT
114	0	CC
115	0	CC
116	0	CC
117	0	CC
118	0	CT
119	0	CC
120	0	CC
121	0	CC
122	0	TT
123	0	CC
124	0	CT
125	0	CC
126	0	CC
127	0	CC
128	0	CT
129	0	CT
130	0	CC
131	0	CT
132	0	CC
133	0	CT
134	0	CC
135	0	CC
136	0	CC
137	0	CC
138	0	CT
139	0	CT

140	0	CT
141	0	CT
142	0	CC
143	0	CC
144	0	CC
145	0	TT
146	0	CC
147	0	CT
148	0	CC
149	0	CT
150	0	CT
151	0	CT
152	0	CC
153	0	CT
154	0	CC
155	0	CT
156	0	CC
157	0	CC
158	0	TT
159	0	CT
160	0	TT
161	0	CC
162	0	CC
163	0	TT
164	0	CC
165	0	CT
166	0	CC
167	0	TT
168	0	CT
169	0	CC
170	0	CT
171	0	CC
172	0	CT
173	0	CT
174	1	CC
175	0	CT
176	0	CT
177	0	CT
178	0	CT
179	0	CT
180	0	CC
181	0	CC
182	0	CT
183	0	CC
184	0	CC or CT
185	1	CT

186	0	CC
187	0	CC
188	1	CT
189	1	CT
190	0	CT
191	1	CC
192	1	CC
193	1	CC or CT
194	0	CC
195	0	CC
196	0	CC
197	0	CT
198	0	CC
199	0	CC
200	1	CC
201	0	CC
202	0	CC
203	0	CT
204	1	CT
205	0	CT
206	0	CC
207	0	CC
208	0	CC
209	0	CC
210	0	CT
211	0	CC
212	1	CC
213	1	CT
214	0	CT
215	1	CT
216	0	CT
217	0	CC
218	1	CT
219	0	CT
220	1	CC
221	0	CC
222	0	TT
223	0	CT
224	0	CC
225	0	CC
226	0	CT
227	0	CT
228	0	CC
229	0	CT
230	0	CC
231	0	CC

232	1	CT
233	0	TT
234	0	TT
235	0	CC
236	1	CC
237	0	CC
238	0	CC
239	0	CT
240	0	CC
241	0	CT
242	0	TT
243	0	CT
244	0	CT
245	0	TT
246	0	CT
247	0	CC
248	0	CT
249	0	TT
250	0	TT
251	0	CT
252	0	CC
253	0	TT
254	0	CT
255	0	CC
256	0	CC
257	0	CC
258	0	CC
259	0	CT
260	0	CT
261	1	CC
262	0	CC
263	0	TT
264	0	CT
265	0	CT
266	0	CT
267	1	CC
268	0	CC
269	0	CT
270	0	CT
271	0	CC
272	0	CT
273	0	TT
274	0	CT
275	0	CT
276	0	CC
277	0	CC

278	0	CC
279	0	CT
280	0	CT
281	0	CT
282	0	CC
283	0	CT
284	0	CT
285	0	CC
286	0	CT
287	0	CT
288	0	CC
289	0	CC
290	1	CC
291	0	CC
292	0	CT
293	0	CT
294	0	CT
295	0	CT
296	0	CC
297	1	CC
298	0	CT
299	0	CT
300	0	CT
301	0	CC
302	0	CT
303	0	CT
304	0	CT
305	0	CT
306	0	CC
307	0	CT
308	1	CC
309	0	CC
310	0	TT
311	0	TT
312	0	CT
313	0	TT
314	0	CC
315	0	CC
316	0	CC
317	0	CT
318	0	CT
319	0	CC
320	0	CT
321	0	CC
322	0	CT
323	0	TT

324	0	CT
325	1	CT
326	0	CC
327	0	CT
328	0	CC
329	0	CT
330	0	CT
331	0	CT
332	1	CT
333	0	CT
334	0	CT
335	0	CT
336	0	CT
337	0	CT
338	0	CT
339	0	CC
340	0	CT
341	0	CT
342	0	CT
343	0	CT
344	0	CT
345	0	TT
346	0	CT
347	0	CT
348	0	CC
349	0	CC
350	0	CC
351	0	TT
352	0	CT
353	0	TT
354	0	CT
355	0	TT
356	0	CT
357	0	CT
358	0	CT
359	0	CT
360	0	CT
361	0	TT
362	0	TT
363	0	CC
364	0	TT
365	0	CT
366	0	CC
367	0	CC
368	0	CT
369	0	CC

370	0	CT
371	0	CT
372	0	TT
373	0	CC
374	0	CC
375	0	CT
376	1	CT
377	0	CC
378	1	TT
379	0	CC
380	0	CC
381	0	CC
382	0	TT
383	0	CC
384	0	CT
385	0	CT
386	0	CT
387	0	CT
388	0	CC
389	0	CT
390	0	CC
391	0	CC
392	0	CT
393	0	CC
394	0	CT
395	0	CT
396	0	CT
397	0	CC
398	0	CT
399	0	CC
400	0	CC
401	0	CC
402	0	CC
403	0	CC
404	0	CT
405	0	TT
406	0	CT
407	0	CT
408	0	CT
409	0	CC
410	0	CT
411	0	CC
412	0	CT
413	0	CT
414	0	CC
415	0	TT

416	0	CC
417	0	CC
418	0	CT
419	0	CC
420	0	CT
421	0	CT
422	0	CC
423	0	CT
424	0	CC
425	0	CT
426	0	CT
427	0	CT
428	0	CT
429	0	CC
430	0	CT
431	0	CT
432	0	CT
433	0	CT
434	0	CC
435	0	CC
436	0	TT
437	0	CT
438	0	CT
439	1	CT
440	0	CT
441	0	TT
442	0	CT
443	0	CC
444	0	CT
445	0	CT
446	0	CC
447	0	CT
448	0	TT
449	0	TT
450	0	CT
451	0	CT
452	0	CT
453	0	CT
454	0	CT
455	0	CC
456	0	CC
457	0	CC
458	0	CT
459	0	CT
460	0	CC
461	0	CC

462	0	CT
463	0	CC
464	0	CT
465	0	CT
466	0	CC
467	0	CT
468	0	CT
469	0	CT
470	0	TT
471	0	CT
472	0	CC
473	0	CT
474	0	CT
475	0	CT
476	0	CC
477	0	CC
478	0	CT
479	0	CC
480	0	CC
481	1	CC
482	0	TT
483	0	CT
484	0	CT
485	0	CT
486	0	CT
487	0	CT
488	0	CT
489	0	CC
490	0	CC
491	0	CT
492	0	CT
493	0	CT
494	0	CT
495	0	CC
496	0	CC
497	0	CT
498	0	CT
499	0	CT
500	0	CC
501	0	CT
502	0	CC
503	0	CC
504	0	TT
505	0	CC
506	0	CC
507	0	TT

508	0	CT
509	0	CC
510	0	CT
511	0	CC
512	0	CT
513	0	CC
514	0	CC
515	0	CT
516	0	CC
517	0	CC
518	0	CC
519	0	CT
520	0	CC
521	0	CC
522	0	CC
523	0	CC
524	0	TT
525	0	CT
526	0	CC
527	0	CT
528	0	CT
529	0	CT
530	0	CC
531	0	CT
532	0	CC
533	0	CC
534	0	CC
535	0	CC
536	0	CT
537	0	CT
538	0	CT
539	0	CT
540	0	CC
541	0	TT
542	0	CT
543	0	CT
544	0	CT
545	0	CT
546	0	TT
547	0	TT
548	0	CC
549	0	CC
550	0	CC
551	0	CC
552	0	TT
553	0	CT

554	0	CC
555	0	CT
556	0	CC
557	0	CC
558	0	CC
559	0	CC
560	0	CC
561	0	CT
562	1	CT
563	0	CT
564	0	CT
565	0	CT
566	0	TT
567	0	CC
568	0	CC
569	0	CC
570	0	CC
571	0	CC
572	0	CC
573	0	CT
574	0	CT
575	0	CT
576	0	CC
577	0	CC
578	0	CT
579	0	CT
580	0	CT
581	0	TT
582	0	CC
583	0	CC
584	0	CT
585	0	CC
586	0	CC
587	0	CT
588	0	CC
589	0	CT
590	0	CC
591	0	CT
592	0	CT
593	0	CC
594	0	CT
595	0	CT
596	1	CT
597	0	CC
598	0	CC
599	0	CT

600	0	CC
601	0	TT
602	0	CT
603	0	CC
604	0	CC
605	0	CT
606	0	CT
607	0	CC
608	0	CT
609	0	CC
611	0	CT
612	0	CT
613	0	CC
614	0	CC
615	0	CC
616	0	CT
617	0	CT
618	0	CT
619	0	CC
620	0	CC
621	0	CC
622	0	CT
623	0	CC
624	0	CC
625	0	CT
626	0	CC
627	0	CC
628	0	CT
629	0	CC
630	0	CC
631	0	CC
632	0	TT
633	0	CC
634	0	CC
635	0	CT
636	0	CT
637	0	CC
638	0	CC
639	0	CT
640	0	TT
641	0	CC
642	0	CC
643	0	CC
644	0	CT
645	0	CT
646	0	CT

647	0	TT
648	0	CT
649	0	TT
650	0	CC
651	0	CC
652	0	CT
653	0	CT
654	0	CT
655	0	TT
656	0	TT
657	0	CT
658	0	CT
659	0	CT
660	0	TT
661	0	CT
662	0	CT
663	0	CT
664	0	CC
665	0	CC
666	0	CC
667	0	CT
668	0	CT
669	0	CT
670	0	CT
671	0	CT
672	0	CT
673	0	CT
674	0	CT
675	0	CT
676	0	CT
677	0	CT
678	0	CT
679	0	CT
680	0	CT
681	0	CT
682	0	CC
683	0	CC
684	0	CT
685	0	CT
686	0	CT
687	0	CT
688	0	CC
689	0	CC
690	0	CT
691	0	CT
692	0	CC

693	0	CT
694	0	CC
695	0	TT
696	0	CT
697	0	CT
698	0	CC
699	0	CC
700	0	CT
701	0	CT
702	0	CT
703	0	CT
704	0	CT
705	0	TT
706	0	CT
707	0	CC
708	0	CT
709	0	CC
710	0	CT
711	0	CT
712	0	CC
713	0	CT
714	0	CC
716	0	TT
717	0	CT
718	0	TT
719	0	CT
720	0	CT
721	0	CT
722	0	CT
723	0	CC
724	0	CT
725	0	CC
726	0	CT
727	0	TT
728	0	CT
729	0	CT
730	0	CC
731	0	CT
732	0	CT
733	0	CT
734	0	CT
735	0	CT
736	0	CT
737	0	CT
738	0	CC
739	0	TT

740	0	CC
741	0	TT
742	0	CC
743	0	CC
744	0	CT
745	0	TT
746	0	CT
747	0	CT
748	0	CT
749	0	CT
750	0	CT
751	0	CT
752	0	CT
753	0	TT
754	0	CT
755	0	CC
756	0	TT
757	0	TT
758	0	CT
759	0	CT
760	0	TT
761	0	CT
762	0	CT
763	0	CT
764	0	CT
765	0	CT
766	0	CC
767	0	CC
768	0	CT
769	0	CC
770	0	CT
771	0	CC
772	0	CC
773	0	TT
774	0	TT
775	0	CT
776	0	CC
777	0	CC
778	0	CT
779	0	CC
780	0	TT
781	0	CC
782	0	CT
783	0	CT
784	0	CC
785	0	CT

786	0	CT
787	0	CT
788	0	CT
789	0	TT
790	0	CT
791	0	CT
792	0	CT
793	0	CT
794	0	CT
795	0	CC
796	0	CC
797	0	CT
798	1	CC
799	0	CT
800	0	CT
801	0	CT
802	0	CC
803	0	CT
804	0	CT
805	0	TT
806	0	CT
807	0	CC
808	0	CT
809	0	CC
810	0	CC
811	0	CC
812	0	CC
813	0	CT
814	0	CC
815	0	CC
816	0	CT
817	1	CT
818	0	CT
819	0	CT
820	0	CC
821	0	CC
822	0	CT
823	0	CT
824	0	CC
825	0	CC
826	0	CC
827	0	CT
828	0	CT
829	0	CC
830	0	CC
831	0	CC

832	0	CT
833	0	CC
834	0	CT
835	0	CT
836	0	CC
837	0	CT
838	0	CC
839	0	CC
840	0	CT
841	0	CC
842	0	CT
843	0	CC
844	0	CT
845	0	TT
846	0	CC
847	0	CC
848	0	CC
849	0	CT
850	0	CC
851	0	CT
852	0	CC
853	0	CT
854	0	CC
855	0	CT
856	0	CC
857	0	CC
858	0	CT
859	0	CT
860	0	CT
861	0	CT
862	0	CC
863	0	CC
864	0	CC
865	0	CC
866	0	CT
867	0	CT
868	0	CT
869	0	CT
870	0	CC
871	0	CT
872	0	CT
873	0	CT
875	0	CC
876	0	CC
877	0	CT
878	1	CC

879	0	TT
880	1	CC
881	0	CC
882	0	CT
883	0	CT
884	0	CC
885	1	CT
886	0	CC
887	0	CC
888	0	CC
889	0	CC
890	0	CC
891	0	CC
892	0	CC
893	0	CC
894	0	CT
895	0	TT
896	0	CC
897	0	CC
898	0	CT
899	1	CC
900	0	CC
901	0	CT
902	0	CT
903	0	CC
904	0	TT
905	0	CC
906	0	CT
907	0	CC
908	0	TT
909	0	CT
910	0	CT
911	0	CT
912	0	CC
913	0	CT
914	1	CC
915	0	CC
916	0	CC
917	0	CT
918	0	CT
919	0	CT
920	0	CT
921	1	CC
922	0	CT
923	1	CC
924	0	CC

925	0	CC
926	0	CT
927	0	CT
928	0	CC
929	0	CC
930	0	CC
931	0	CC
932	0	CC
933	0	CT
934	1	CT
935	0	CT
936	0	CC
937	0	CT
938	0	CC
939	0	CT
940	0	CT
941	0	CT
942	0	CC
943	0	CT
944	0	CC
945	0	CT
946	0	CC
947	0	CC
948	0	CC
949	0	CT
950	0	CT
951	0	CT
952	0	CT
953	0	CC
954	0	CT
955	0	CC
956	0	CT
957	0	CC
958	0	CC
959	1	CC
960	0	CC
961	0	CT
962	1	CC
963	0	CC
964	0	CT
965	0	CT
966	0	CC
967	0	CC
968	0	CT
969	1	CT
970	0	CC

971	0	CC
972	0	CC
973	0	CC
974	0	CC
975	0	CT
976	0	TT
977	0	TT
978	0	CC
979	0	CC

No – number of cattle
BHB – 1 sick cows; 0 healthy cows

Statistical Analysis ***(data analysis and visualization program by STATISTICA®13)***

The odds ratio was used to find the probability of an outcome of an event which there were three possible outcomes and there is a plausible causal effect.

The function was defined by the following formula:

$$Odds\,Ratio = \frac{P_x/(1-P_x)}{P_y/¿¿}$$

Where:

Px - the probability of default with low income

(1-Px) - the probability of non-default with low income

Py - the probability of default with high income

(1-Py) – the probability of non-default with high income.

Parameter estimation; Modeled probability BHB0/1 = 0

Effect	Standard error	upper 95,0%	lower 95,0%
free expression	1,828±0.20	-1,268	5.897
milk(kg)	0.012±0.00*	-0.079	-0.028
fat(%)	0.177±0.00*	-1.02155	-0.325
pro(%)	0.466±0.00*	0.54118	2.367

Statistically significant differences $p < 0.05$*

Odds ratio; Modeled probability BHB0/1 = 0

Effect	Odds ratio	upper 95,0%	lower 95,0%	*p-value*
milk(kg)	0.947	0.923	0.971	0.000
fat(%)	0.509	0.360	0.721	0.000

pro(%)	4.282	1.718	10.675	0.001

Statistically significant differences $p < 0.05$

ROC (Receiver Operating Characteristics) curve as an evaluation metrics was choose for checking classification models performance for BHB0/1 and OPN.

The function was defined by the following formula:

$$Sensitivity = \frac{True\ positive}{True\ positive + False\ negative}$$

$$Specificity = \frac{True\ negative}{True\ negative + False\ positive}$$

Sensitivity and specificity, cutoff point for all data set (n=979)

No.	Fitting of ROC; binding: Modeled probability BHB0/1 = 0						
	Cutoff point	True positive	True negative	False negative	False positive	Sensitivity	1- Specificity
1	0,996863	1,0000	50,00000	0,00000	923,0000	0,001082	0,000000
2	0,996625	2,0000	50,00000	0,00000	922,0000	0,002165	0,000000
3	0,996119	3,0000	50,00000	0,00000	921,0000	0,003247	0,000000
4	0,995672	4,0000	50,00000	0,00000	920,0000	0,004329	0,000000
5	0,995158	5,0000	50,00000	0,00000	919,0000	0,005411	0,000000
6	0,995154	6,0000	50,00000	0,00000	918,0000	0,006494	0,000000
7	0,995068	7,0000	50,00000	0,00000	917,0000	0,007576	0,000000
8	0,995012	8,0000	50,00000	0,00000	916,0000	0,008658	0,000000
9	0,994940	9,0000	50,00000	0,00000	915,0000	0,009740	0,000000
10	0,994657	10,0000	50,00000	0,00000	914,0000	0,010823	0,000000
11	0,994630	11,0000	50,00000	0,00000	913,0000	0,011905	0,000000
12	0,994522	12,0000	50,00000	0,00000	912,0000	0,012987	0,000000
13	0,994437	13,0000	50,00000	0,00000	911,0000	0,014069	0,000000
14	0,994420	14,0000	50,00000	0,00000	910,0000	0,015152	0,000000
15	0,994221	15,0000	50,00000	0,00000	909,0000	0,016234	0,000000
16	0,994172	16,0000	50,00000	0,00000	908,0000	0,017316	0,000000
17	0,994120	17,0000	50,00000	0,00000	907,0000	0,018398	0,000000
18	0,993998	18,0000	50,00000	0,00000	906,0000	0,019481	0,000000
19	0,993736	19,0000	50,00000	0,00000	905,0000	0,020563	0,000000
20	0,993613	20,0000	50,00000	0,00000	904,0000	0,021645	0,000000
21	0,993243	21,0000	50,00000	0,00000	903,0000	0,022727	0,000000
22	0,993225	22,0000	50,00000	0,00000	902,0000	0,023810	0,000000
23	0,993047	23,0000	50,00000	0,00000	901,0000	0,024892	0,000000
24	0,992975	24,0000	50,00000	0,00000	900,0000	0,025974	0,000000
25	0,992895	25,0000	50,00000	0,00000	899,0000	0,027056	0,000000
26	0,992887	26,0000	50,00000	0,00000	898,0000	0,028139	0,000000

27	0,992795	27,0000	50,00000	0,00000	897,0000	0,029221	0,000000
28	0,992658	28,0000	50,00000	0,00000	896,0000	0,030303	0,000000
29	0,992605	29,0000	50,00000	0,00000	895,0000	0,031385	0,000000
30	0,992601	30,0000	50,00000	0,00000	894,0000	0,032468	0,000000
31	0,992593	31,0000	50,00000	0,00000	893,0000	0,033550	0,000000
32	0,992520	32,0000	50,00000	0,00000	892,0000	0,034632	0,000000
33	0,992491	33,0000	50,00000	0,00000	891,0000	0,035714	0,000000
34	0,992488	34,0000	50,00000	0,00000	890,0000	0,036797	0,000000
35	0,992305	35,0000	50,00000	0,00000	889,0000	0,037879	0,000000
36	0,992270	36,0000	50,00000	0,00000	888,0000	0,038961	0,000000
37	0,992265	37,0000	50,00000	0,00000	887,0000	0,040043	0,000000
38	0,992204	38,0000	50,00000	0,00000	886,0000	0,041126	0,000000
39	0,992076	39,0000	50,00000	0,00000	885,0000	0,042208	0,000000
40	0,991918	40,0000	50,00000	0,00000	884,0000	0,043290	0,000000
41	0,991874	41,0000	50,00000	0,00000	883,0000	0,044372	0,000000
42	0,991842	42,0000	50,00000	0,00000	882,0000	0,045455	0,000000
43	0,991834	43,0000	50,00000	0,00000	881,0000	0,046537	0,000000
44	0,991782	44,0000	50,00000	0,00000	880,0000	0,047619	0,000000
45	0,991548	45,0000	50,00000	0,00000	879,0000	0,048701	0,000000
46	0,991529	46,0000	50,00000	0,00000	878,0000	0,049784	0,000000
47	0,991494	47,0000	50,00000	0,00000	877,0000	0,050866	0,000000
48	0,991381	48,0000	50,00000	0,00000	876,0000	0,051948	0,000000
49	0,991325	49,0000	50,00000	0,00000	875,0000	0,053030	0,000000
50	0,991020	50,0000	50,00000	0,00000	874,0000	0,054113	0,000000
51	0,991000	51,0000	50,00000	0,00000	873,0000	0,055195	0,000000
52	0,990964	52,0000	50,00000	0,00000	872,0000	0,056277	0,000000
53	0,990934	53,0000	50,00000	0,00000	871,0000	0,057359	0,000000
54	0,990857	53,0000	49,00000	1,00000	871,0000	0,057359	0,020000
55	0,990721	54,0000	49,00000	1,00000	870,0000	0,058442	0,020000
56	0,990690	55,0000	49,00000	1,00000	869,0000	0,059524	0,020000
57	0,990678	56,0000	49,00000	1,00000	868,0000	0,060606	0,020000
58	0,990633	57,0000	49,00000	1,00000	867,0000	0,061688	0,020000
59	0,990627	58,0000	49,00000	1,00000	866,0000	0,062771	0,020000
60	0,990580	59,0000	49,00000	1,00000	865,0000	0,063853	0,020000
61	0,990524	60,0000	49,00000	1,00000	864,0000	0,064935	0,020000
62	0,990375	61,0000	49,00000	1,00000	863,0000	0,066017	0,020000
63	0,990349	62,0000	49,00000	1,00000	862,0000	0,067100	0,020000
64	0,990294	63,0000	49,00000	1,00000	861,0000	0,068182	0,020000
65	0,990185	64,0000	49,00000	1,00000	860,0000	0,069264	0,020000
66	0,990174	65,0000	49,00000	1,00000	859,0000	0,070346	0,020000
67	0,990158	66,0000	49,00000	1,00000	858,0000	0,071429	0,020000
68	0,990082	67,0000	49,00000	1,00000	857,0000	0,072511	0,020000
69	0,990052	68,0000	49,00000	1,00000	856,0000	0,073593	0,020000
70	0,990007	69,0000	49,00000	1,00000	855,0000	0,074675	0,020000
71	0,989972	70,0000	49,00000	1,00000	854,0000	0,075758	0,020000
72	0,989937	71,0000	49,00000	1,00000	853,0000	0,076840	0,020000

73	0,989773	72,0000	49,00000	1,00000	852,0000	0,077922	0,020000
74	0,989738	73,0000	49,00000	1,00000	851,0000	0,079004	0,020000
75	0,989583	74,0000	49,00000	1,00000	850,0000	0,080087	0,020000
76	0,989576	75,0000	49,00000	1,00000	849,0000	0,081169	0,020000
77	0,989549	76,0000	49,00000	1,00000	848,0000	0,082251	0,020000
78	0,989546	77,0000	49,00000	1,00000	847,0000	0,083333	0,020000
79	0,989403	78,0000	49,00000	1,00000	846,0000	0,084416	0,020000
80	0,989379	79,0000	49,00000	1,00000	845,0000	0,085498	0,020000
81	0,989368	80,0000	49,00000	1,00000	844,0000	0,086580	0,020000
82	0,989305	81,0000	49,00000	1,00000	843,0000	0,087662	0,020000
83	0,989252	82,0000	49,00000	1,00000	842,0000	0,088745	0,020000
84	0,989204	83,0000	49,00000	1,00000	841,0000	0,089827	0,020000
85	0,989124	84,0000	49,00000	1,00000	840,0000	0,090909	0,020000
86	0,989090	85,0000	49,00000	1,00000	839,0000	0,091991	0,020000
87	0,989079	86,0000	49,00000	1,00000	838,0000	0,093074	0,020000
88	0,988944	87,0000	49,00000	1,00000	837,0000	0,094156	0,020000
89	0,988927	88,0000	49,00000	1,00000	836,0000	0,095238	0,020000
90	0,988912	89,0000	49,00000	1,00000	835,0000	0,096320	0,020000
91	0,988827	90,0000	49,00000	1,00000	834,0000	0,097403	0,020000
92	0,988803	91,0000	49,00000	1,00000	833,0000	0,098485	0,020000
93	0,988778	92,0000	49,00000	1,00000	832,0000	0,099567	0,020000
94	0,988693	92,0000	48,00000	2,00000	832,0000	0,099567	0,040000
95	0,988650	93,0000	48,00000	2,00000	831,0000	0,100649	0,040000
96	0,988610	94,0000	48,00000	2,00000	830,0000	0,101732	0,040000
97	0,988576	95,0000	48,00000	2,00000	829,0000	0,102814	0,040000
98	0,988518	96,0000	48,00000	2,00000	828,0000	0,103896	0,040000
99	0,988512	97,0000	48,00000	2,00000	827,0000	0,104978	0,040000
100	0,988494	98,0000	48,00000	2,00000	826,0000	0,106061	0,040000
101	0,988431	99,0000	48,00000	2,00000	825,0000	0,107143	0,040000
102	0,988406	100,0000	48,00000	2,00000	824,0000	0,108225	0,040000
103	0,988389	101,0000	48,00000	2,00000	823,0000	0,109307	0,040000
104	0,988357	102,0000	48,00000	2,00000	822,0000	0,110390	0,040000
105	0,988322	103,0000	48,00000	2,00000	821,0000	0,111472	0,040000
106	0,988315	104,0000	48,00000	2,00000	820,0000	0,112554	0,040000
107	0,988253	105,0000	48,00000	2,00000	819,0000	0,113636	0,040000
108	0,988201	106,0000	48,00000	2,00000	818,0000	0,114719	0,040000
109	0,988191	107,0000	48,00000	2,00000	817,0000	0,115801	0,040000
110	0,988123	108,0000	48,00000	2,00000	816,0000	0,116883	0,040000
111	0,988123	109,0000	48,00000	2,00000	815,0000	0,117965	0,040000
112	0,988102	110,0000	48,00000	2,00000	814,0000	0,119048	0,040000
113	0,988095	111,0000	48,00000	2,00000	813,0000	0,120130	0,040000
114	0,987800	112,0000	48,00000	2,00000	812,0000	0,121212	0,040000
115	0,987767	113,0000	48,00000	2,00000	811,0000	0,122294	0,040000
116	0,987712	114,0000	48,00000	2,00000	810,0000	0,123377	0,040000
117	0,987682	115,0000	48,00000	2,00000	809,0000	0,124459	0,040000
118	0,987601	116,0000	48,00000	2,00000	808,0000	0,125541	0,040000

119	0,987597	117,0000	48,00000	2,00000	807,0000	0,126623	0,040000
120	0,987565	118,0000	48,00000	2,00000	806,0000	0,127706	0,040000
121	0,987536	119,0000	48,00000	2,00000	805,0000	0,128788	0,040000
122	0,987451	120,0000	48,00000	2,00000	804,0000	0,129870	0,040000
123	0,987390	121,0000	48,00000	2,00000	803,0000	0,130952	0,040000
124	0,987316	122,0000	48,00000	2,00000	802,0000	0,132035	0,040000
125	0,987243	123,0000	48,00000	2,00000	801,0000	0,133117	0,040000
126	0,987220	124,0000	48,00000	2,00000	800,0000	0,134199	0,040000
127	0,987213	125,0000	48,00000	2,00000	799,0000	0,135281	0,040000
128	0,987197	126,0000	48,00000	2,00000	798,0000	0,136364	0,040000
129	0,986887	127,0000	48,00000	2,00000	797,0000	0,137446	0,040000
130	0,986839	128,0000	48,00000	2,00000	796,0000	0,138528	0,040000
131	0,986808	129,0000	48,00000	2,00000	795,0000	0,139610	0,040000
132	0,986746	130,0000	48,00000	2,00000	794,0000	0,140693	0,040000
133	0,986669	131,0000	48,00000	2,00000	793,0000	0,141775	0,040000
134	0,986563	132,0000	48,00000	2,00000	792,0000	0,142857	0,040000
135	0,986542	133,0000	48,00000	2,00000	791,0000	0,143939	0,040000
136	0,986531	134,0000	48,00000	2,00000	790,0000	0,145022	0,040000
137	0,986470	135,0000	48,00000	2,00000	789,0000	0,146104	0,040000
138	0,986442	136,0000	48,00000	2,00000	788,0000	0,147186	0,040000
139	0,986411	137,0000	48,00000	2,00000	787,0000	0,148268	0,040000
140	0,986402	138,0000	48,00000	2,00000	786,0000	0,149351	0,040000
141	0,986350	139,0000	48,00000	2,00000	785,0000	0,150433	0,040000
142	0,986349	140,0000	48,00000	2,00000	784,0000	0,151515	0,040000
143	0,986346	140,0000	47,00000	3,00000	784,0000	0,151515	0,060000
144	0,986273	141,0000	47,00000	3,00000	783,0000	0,152597	0,060000
145	0,986197	142,0000	47,00000	3,00000	782,0000	0,153680	0,060000
146	0,986184	143,0000	47,00000	3,00000	781,0000	0,154762	0,060000
147	0,986153	144,0000	47,00000	3,00000	780,0000	0,155844	0,060000
148	0,986056	145,0000	47,00000	3,00000	779,0000	0,156926	0,060000
149	0,986039	146,0000	47,00000	3,00000	778,0000	0,158009	0,060000
150	0,985934	147,0000	47,00000	3,00000	777,0000	0,159091	0,060000
151	0,985903	148,0000	47,00000	3,00000	776,0000	0,160173	0,060000
152	0,985888	149,0000	47,00000	3,00000	775,0000	0,161255	0,060000
153	0,985887	150,0000	47,00000	3,00000	774,0000	0,162338	0,060000
154	0,985874	151,0000	47,00000	3,00000	773,0000	0,163420	0,060000
155	0,985793	152,0000	47,00000	3,00000	772,0000	0,164502	0,060000
156	0,985574	153,0000	47,00000	3,00000	771,0000	0,165584	0,060000
157	0,985560	154,0000	47,00000	3,00000	770,0000	0,166667	0,060000
158	0,985504	155,0000	47,00000	3,00000	769,0000	0,167749	0,060000
159	0,985486	156,0000	47,00000	3,00000	768,0000	0,168831	0,060000
160	0,985384	157,0000	47,00000	3,00000	767,0000	0,169913	0,060000
161	0,985368	158,0000	47,00000	3,00000	766,0000	0,170996	0,060000
162	0,985322	159,0000	47,00000	3,00000	765,0000	0,172078	0,060000
163	0,985312	160,0000	47,00000	3,00000	764,0000	0,173160	0,060000
164	0,985093	161,0000	47,00000	3,00000	763,0000	0,174242	0,060000

165	0,985071	162,0000	47,00000	3,00000	762,0000	0,175325	0,060000
166	0,985047	163,0000	47,00000	3,00000	761,0000	0,176407	0,060000
167	0,985034	164,0000	47,00000	3,00000	760,0000	0,177489	0,060000
168	0,984974	165,0000	47,00000	3,00000	759,0000	0,178571	0,060000
169	0,984777	166,0000	47,00000	3,00000	758,0000	0,179654	0,060000
170	0,984758	167,0000	47,00000	3,00000	757,0000	0,180736	0,060000
171	0,984756	168,0000	47,00000	3,00000	756,0000	0,181818	0,060000
172	0,984728	169,0000	47,00000	3,00000	755,0000	0,182900	0,060000
173	0,984708	170,0000	47,00000	3,00000	754,0000	0,183983	0,060000
174	0,984548	171,0000	47,00000	3,00000	753,0000	0,185065	0,060000
175	0,984446	172,0000	47,00000	3,00000	752,0000	0,186147	0,060000
176	0,984422	173,0000	47,00000	3,00000	751,0000	0,187229	0,060000
177	0,984413	173,0000	46,00000	4,00000	751,0000	0,187229	0,080000
178	0,984410	174,0000	46,00000	4,00000	750,0000	0,188312	0,080000
179	0,984352	175,0000	46,00000	4,00000	749,0000	0,189394	0,080000
180	0,984320	176,0000	46,00000	4,00000	748,0000	0,190476	0,080000
181	0,984230	177,0000	46,00000	4,00000	747,0000	0,191558	0,080000
182	0,984229	178,0000	46,00000	4,00000	746,0000	0,192641	0,080000
183	0,984194	179,0000	46,00000	4,00000	745,0000	0,193723	0,080000
184	0,984078	180,0000	46,00000	4,00000	744,0000	0,194805	0,080000
185	0,984020	181,0000	46,00000	4,00000	743,0000	0,195887	0,080000
186	0,983977	182,0000	46,00000	4,00000	742,0000	0,196970	0,080000
187	0,983926	183,0000	46,00000	4,00000	741,0000	0,198052	0,080000
188	0,983906	184,0000	46,00000	4,00000	740,0000	0,199134	0,080000
189	0,983850	185,0000	46,00000	4,00000	739,0000	0,200216	0,080000
190	0,983726	186,0000	46,00000	4,00000	738,0000	0,201299	0,080000
191	0,983704	187,0000	46,00000	4,00000	737,0000	0,202381	0,080000
192	0,983698	188,0000	46,00000	4,00000	736,0000	0,203463	0,080000
193	0,983681	189,0000	46,00000	4,00000	735,0000	0,204545	0,080000
194	0,983660	190,0000	46,00000	4,00000	734,0000	0,205628	0,080000
195	0,983618	191,0000	46,00000	4,00000	733,0000	0,206710	0,080000
196	0,983559	192,0000	46,00000	4,00000	732,0000	0,207792	0,080000
197	0,983535	193,0000	46,00000	4,00000	731,0000	0,208874	0,080000
198	0,983520	194,0000	46,00000	4,00000	730,0000	0,209957	0,080000
199	0,983507	195,0000	46,00000	4,00000	729,0000	0,211039	0,080000
200	0,983485	196,0000	46,00000	4,00000	728,0000	0,212121	0,080000
201	0,983429	197,0000	46,00000	4,00000	727,0000	0,213203	0,080000
202	0,983370	198,0000	46,00000	4,00000	726,0000	0,214286	0,080000
203	0,983321	199,0000	46,00000	4,00000	725,0000	0,215368	0,080000
204	0,983264	200,0000	46,00000	4,00000	724,0000	0,216450	0,080000
205	0,983213	201,0000	46,00000	4,00000	723,0000	0,217532	0,080000
206	0,983116	202,0000	46,00000	4,00000	722,0000	0,218615	0,080000
207	0,983071	203,0000	46,00000	4,00000	721,0000	0,219697	0,080000
208	0,982937	204,0000	46,00000	4,00000	720,0000	0,220779	0,080000
209	0,982891	205,0000	46,00000	4,00000	719,0000	0,221861	0,080000
210	0,982884	206,0000	46,00000	4,00000	718,0000	0,222944	0,080000

211	0,982871	207,0000	46,00000	4,00000	717,0000	0,224026	0,080000
212	0,982869	208,0000	46,00000	4,00000	716,0000	0,225108	0,080000
213	0,982854	209,0000	46,00000	4,00000	715,0000	0,226190	0,080000
214	0,982843	210,0000	46,00000	4,00000	714,0000	0,227273	0,080000
215	0,982840	211,0000	46,00000	4,00000	713,0000	0,228355	0,080000
216	0,982817	212,0000	46,00000	4,00000	712,0000	0,229437	0,080000
217	0,982770	213,0000	46,00000	4,00000	711,0000	0,230519	0,080000
218	0,982627	214,0000	46,00000	4,00000	710,0000	0,231602	0,080000
219	0,982564	215,0000	46,00000	4,00000	709,0000	0,232684	0,080000
220	0,982418	216,0000	46,00000	4,00000	708,0000	0,233766	0,080000
221	0,982410	217,0000	46,00000	4,00000	707,0000	0,234848	0,080000
222	0,982398	218,0000	46,00000	4,00000	706,0000	0,235931	0,080000
223	0,982327	219,0000	46,00000	4,00000	705,0000	0,237013	0,080000
224	0,982268	220,0000	46,00000	4,00000	704,0000	0,238095	0,080000
225	0,982267	221,0000	46,00000	4,00000	703,0000	0,239177	0,080000
226	0,982228	222,0000	46,00000	4,00000	702,0000	0,240260	0,080000
227	0,982221	223,0000	46,00000	4,00000	701,0000	0,241342	0,080000
228	0,982166	224,0000	46,00000	4,00000	700,0000	0,242424	0,080000
229	0,982116	225,0000	46,00000	4,00000	699,0000	0,243506	0,080000
230	0,982091	226,0000	46,00000	4,00000	698,0000	0,244589	0,080000
231	0,981930	227,0000	46,00000	4,00000	697,0000	0,245671	0,080000
232	0,981929	228,0000	46,00000	4,00000	696,0000	0,246753	0,080000
233	0,981822	229,0000	46,00000	4,00000	695,0000	0,247835	0,080000
234	0,981808	230,0000	46,00000	4,00000	694,0000	0,248918	0,080000
235	0,981729	231,0000	46,00000	4,00000	693,0000	0,250000	0,080000
236	0,981496	232,0000	46,00000	4,00000	692,0000	0,251082	0,080000
237	0,981406	233,0000	46,00000	4,00000	691,0000	0,252165	0,080000
238	0,981380	234,0000	46,00000	4,00000	690,0000	0,253247	0,080000
239	0,981270	235,0000	46,00000	4,00000	689,0000	0,254329	0,080000
240	0,981058	236,0000	46,00000	4,00000	688,0000	0,255411	0,080000
241	0,980935	237,0000	46,00000	4,00000	687,0000	0,256494	0,080000
242	0,980880	238,0000	46,00000	4,00000	686,0000	0,257576	0,080000
243	0,980875	239,0000	46,00000	4,00000	685,0000	0,258658	0,080000
244	0,980833	240,0000	46,00000	4,00000	684,0000	0,259740	0,080000
245	0,980812	241,0000	46,00000	4,00000	683,0000	0,260823	0,080000
246	0,980703	242,0000	46,00000	4,00000	682,0000	0,261905	0,080000
247	0,980667	243,0000	46,00000	4,00000	681,0000	0,262987	0,080000
248	0,980654	244,0000	46,00000	4,00000	680,0000	0,264069	0,080000
249	0,980619	245,0000	46,00000	4,00000	679,0000	0,265152	0,080000
250	0,980590	246,0000	46,00000	4,00000	678,0000	0,266234	0,080000
251	0,980377	247,0000	46,00000	4,00000	677,0000	0,267316	0,080000
252	0,980358	248,0000	46,00000	4,00000	676,0000	0,268398	0,080000
253	0,980324	249,0000	46,00000	4,00000	675,0000	0,269481	0,080000
254	0,980273	250,0000	46,00000	4,00000	674,0000	0,270563	0,080000
255	0,980127	251,0000	46,00000	4,00000	673,0000	0,271645	0,080000
256	0,980099	252,0000	46,00000	4,00000	672,0000	0,272727	0,080000

257	0,980015	254,0000	46,00000	4,00000	670,0000	0,274892	0,080000
258	0,979976	255,0000	46,00000	4,00000	669,0000	0,275974	0,080000
259	0,979805	256,0000	46,00000	4,00000	668,0000	0,277056	0,080000
260	0,979739	257,0000	46,00000	4,00000	667,0000	0,278139	0,080000
261	0,979734	258,0000	46,00000	4,00000	666,0000	0,279221	0,080000
262	0,979719	259,0000	46,00000	4,00000	665,0000	0,280303	0,080000
263	0,979633	260,0000	46,00000	4,00000	664,0000	0,281385	0,080000
264	0,979604	261,0000	46,00000	4,00000	663,0000	0,282468	0,080000
265	0,979581	262,0000	46,00000	4,00000	662,0000	0,283550	0,080000
266	0,979572	263,0000	46,00000	4,00000	661,0000	0,284632	0,080000
267	0,979532	264,0000	46,00000	4,00000	660,0000	0,285714	0,080000
268	0,979479	265,0000	46,00000	4,00000	659,0000	0,286797	0,080000
269	0,979382	266,0000	46,00000	4,00000	658,0000	0,287879	0,080000
270	0,979374	267,0000	46,00000	4,00000	657,0000	0,288961	0,080000
271	0,979227	268,0000	46,00000	4,00000	656,0000	0,290043	0,080000
272	0,979059	269,0000	46,00000	4,00000	655,0000	0,291126	0,080000
273	0,979016	269,0000	45,00000	5,00000	655,0000	0,291126	0,100000
274	0,978901	270,0000	45,00000	5,00000	654,0000	0,292208	0,100000
275	0,978883	271,0000	45,00000	5,00000	653,0000	0,293290	0,100000
276	0,978780	272,0000	45,00000	5,00000	652,0000	0,294372	0,100000
277	0,978764	273,0000	45,00000	5,00000	651,0000	0,295455	0,100000
278	0,978752	274,0000	45,00000	5,00000	650,0000	0,296537	0,100000
279	0,978702	275,0000	45,00000	5,00000	649,0000	0,297619	0,100000
280	0,978697	276,0000	45,00000	5,00000	648,0000	0,298701	0,100000
281	0,978554	277,0000	45,00000	5,00000	647,0000	0,299784	0,100000
282	0,978549	278,0000	45,00000	5,00000	646,0000	0,300866	0,100000
283	0,978413	279,0000	45,00000	5,00000	645,0000	0,301948	0,100000
284	0,978400	280,0000	45,00000	5,00000	644,0000	0,303030	0,100000
285	0,978393	281,0000	45,00000	5,00000	643,0000	0,304113	0,100000
286	0,978265	282,0000	45,00000	5,00000	642,0000	0,305195	0,100000
287	0,978173	283,0000	45,00000	5,00000	641,0000	0,306277	0,100000
288	0,978169	284,0000	45,00000	5,00000	640,0000	0,307359	0,100000
289	0,978125	285,0000	45,00000	5,00000	639,0000	0,308442	0,100000
290	0,978015	286,0000	45,00000	5,00000	638,0000	0,309524	0,100000
291	0,978012	287,0000	45,00000	5,00000	637,0000	0,310606	0,100000
292	0,978008	288,0000	45,00000	5,00000	636,0000	0,311688	0,100000
293	0,977945	289,0000	45,00000	5,00000	635,0000	0,312771	0,100000
294	0,977798	290,0000	45,00000	5,00000	634,0000	0,313853	0,100000
295	0,977714	291,0000	45,00000	5,00000	633,0000	0,314935	0,100000
296	0,977679	292,0000	45,00000	5,00000	632,0000	0,316017	0,100000
297	0,977629	293,0000	45,00000	5,00000	631,0000	0,317100	0,100000
298	0,977521	294,0000	45,00000	5,00000	630,0000	0,318182	0,100000
299	0,977424	295,0000	45,00000	5,00000	629,0000	0,319264	0,100000
300	0,977405	296,0000	45,00000	5,00000	628,0000	0,320346	0,100000
301	0,977395	297,0000	45,00000	5,00000	627,0000	0,321429	0,100000
302	0,977299	298,0000	45,00000	5,00000	626,0000	0,322511	0,100000

303	0,977270	299,0000	45,00000	5,00000	625,0000	0,323593	0,100000
304	0,977262	300,0000	45,00000	5,00000	624,0000	0,324675	0,100000
305	0,977247	301,0000	45,00000	5,00000	623,0000	0,325758	0,100000
306	0,977157	302,0000	45,00000	5,00000	622,0000	0,326840	0,100000
307	0,977106	303,0000	45,00000	5,00000	621,0000	0,327922	0,100000
308	0,977065	304,0000	45,00000	5,00000	620,0000	0,329004	0,100000
309	0,977015	305,0000	45,00000	5,00000	619,0000	0,330087	0,100000
310	0,976966	306,0000	45,00000	5,00000	618,0000	0,331169	0,100000
311	0,976857	307,0000	45,00000	5,00000	617,0000	0,332251	0,100000
312	0,976813	308,0000	45,00000	5,00000	616,0000	0,333333	0,100000
313	0,976729	309,0000	45,00000	5,00000	615,0000	0,334416	0,100000
314	0,976720	310,0000	45,00000	5,00000	614,0000	0,335498	0,100000
315	0,976639	311,0000	45,00000	5,00000	613,0000	0,336580	0,100000
316	0,976635	312,0000	45,00000	5,00000	612,0000	0,337662	0,100000
317	0,976524	313,0000	45,00000	5,00000	611,0000	0,338745	0,100000
318	0,976510	314,0000	45,00000	5,00000	610,0000	0,339827	0,100000
319	0,976492	315,0000	45,00000	5,00000	609,0000	0,340909	0,100000
320	0,976477	316,0000	45,00000	5,00000	608,0000	0,341991	0,100000
321	0,976299	317,0000	45,00000	5,00000	607,0000	0,343074	0,100000
322	0,976220	318,0000	45,00000	5,00000	606,0000	0,344156	0,100000
323	0,976168	319,0000	45,00000	5,00000	605,0000	0,345238	0,100000
324	0,976167	320,0000	45,00000	5,00000	604,0000	0,346320	0,100000
325	0,976098	321,0000	45,00000	5,00000	603,0000	0,347403	0,100000
326	0,976066	322,0000	45,00000	5,00000	602,0000	0,348485	0,100000
327	0,976023	323,0000	45,00000	5,00000	601,0000	0,349567	0,100000
328	0,976015	324,0000	45,00000	5,00000	600,0000	0,350649	0,100000
329	0,976007	324,0000	44,00000	6,00000	600,0000	0,350649	0,120000
330	0,975997	325,0000	44,00000	6,00000	599,0000	0,351732	0,120000
331	0,975905	326,0000	44,00000	6,00000	598,0000	0,352814	0,120000
332	0,975834	327,0000	44,00000	6,00000	597,0000	0,353896	0,120000
333	0,975787	328,0000	44,00000	6,00000	596,0000	0,354978	0,120000
334	0,975716	329,0000	44,00000	6,00000	595,0000	0,356061	0,120000
335	0,975580	330,0000	44,00000	6,00000	594,0000	0,357143	0,120000
336	0,975331	331,0000	44,00000	6,00000	593,0000	0,358225	0,120000
337	0,975160	332,0000	44,00000	6,00000	592,0000	0,359307	0,120000
338	0,975109	333,0000	44,00000	6,00000	591,0000	0,360390	0,120000
339	0,975054	334,0000	44,00000	6,00000	590,0000	0,361472	0,120000
340	0,975005	335,0000	44,00000	6,00000	589,0000	0,362554	0,120000
341	0,974822	336,0000	44,00000	6,00000	588,0000	0,363636	0,120000
342	0,974808	337,0000	44,00000	6,00000	587,0000	0,364719	0,120000
343	0,974694	338,0000	44,00000	6,00000	586,0000	0,365801	0,120000
344	0,974635	339,0000	44,00000	6,00000	585,0000	0,366883	0,120000
345	0,974628	340,0000	44,00000	6,00000	584,0000	0,367965	0,120000
346	0,974486	341,0000	44,00000	6,00000	583,0000	0,369048	0,120000
347	0,974359	341,0000	43,00000	7,00000	583,0000	0,369048	0,140000
348	0,974337	342,0000	43,00000	7,00000	582,0000	0,370130	0,140000

349	0,974266	343,0000	43,00000	7,00000	581,0000	0,371212	0,140000
350	0,974261	344,0000	43,00000	7,00000	580,0000	0,372294	0,140000
351	0,974215	345,0000	43,00000	7,00000	579,0000	0,373377	0,140000
352	0,974161	346,0000	43,00000	7,00000	578,0000	0,374459	0,140000
353	0,974133	347,0000	43,00000	7,00000	577,0000	0,375541	0,140000
354	0,974119	348,0000	43,00000	7,00000	576,0000	0,376623	0,140000
355	0,974019	349,0000	43,00000	7,00000	575,0000	0,377706	0,140000
356	0,973920	350,0000	43,00000	7,00000	574,0000	0,378788	0,140000
357	0,973791	351,0000	43,00000	7,00000	573,0000	0,379870	0,140000
358	0,973775	352,0000	43,00000	7,00000	572,0000	0,380952	0,140000
359	0,973682	353,0000	43,00000	7,00000	571,0000	0,382035	0,140000
360	0,973607	354,0000	43,00000	7,00000	570,0000	0,383117	0,140000
361	0,973596	355,0000	43,00000	7,00000	569,0000	0,384199	0,140000
362	0,973584	356,0000	43,00000	7,00000	568,0000	0,385281	0,140000
363	0,973556	357,0000	43,00000	7,00000	567,0000	0,386364	0,140000
364	0,973465	358,0000	43,00000	7,00000	566,0000	0,387446	0,140000
365	0,973346	359,0000	43,00000	7,00000	565,0000	0,388528	0,140000
366	0,973227	360,0000	43,00000	7,00000	564,0000	0,389610	0,140000
367	0,973177	361,0000	43,00000	7,00000	563,0000	0,390693	0,140000
368	0,973132	362,0000	43,00000	7,00000	562,0000	0,391775	0,140000
369	0,973080	363,0000	43,00000	7,00000	561,0000	0,392857	0,140000
370	0,973058	364,0000	43,00000	7,00000	560,0000	0,393939	0,140000
371	0,972996	365,0000	43,00000	7,00000	559,0000	0,395022	0,140000
372	0,972995	366,0000	43,00000	7,00000	558,0000	0,396104	0,140000
373	0,972975	367,0000	43,00000	7,00000	557,0000	0,397186	0,140000
374	0,972915	368,0000	43,00000	7,00000	556,0000	0,398268	0,140000
375	0,972890	369,0000	43,00000	7,00000	555,0000	0,399351	0,140000
376	0,972809	370,0000	43,00000	7,00000	554,0000	0,400433	0,140000
377	0,972626	371,0000	43,00000	7,00000	553,0000	0,401515	0,140000
378	0,972609	372,0000	43,00000	7,00000	552,0000	0,402597	0,140000
379	0,972571	373,0000	43,00000	7,00000	551,0000	0,403680	0,140000
380	0,972525	374,0000	43,00000	7,00000	550,0000	0,404762	0,140000
381	0,972473	375,0000	43,00000	7,00000	549,0000	0,405844	0,140000
382	0,972469	376,0000	43,00000	7,00000	548,0000	0,406926	0,140000
383	0,972468	377,0000	43,00000	7,00000	547,0000	0,408009	0,140000
384	0,972457	378,0000	43,00000	7,00000	546,0000	0,409091	0,140000
385	0,972372	379,0000	43,00000	7,00000	545,0000	0,410173	0,140000
386	0,972350	380,0000	43,00000	7,00000	544,0000	0,411255	0,140000
387	0,972332	381,0000	43,00000	7,00000	543,0000	0,412338	0,140000
388	0,972303	382,0000	43,00000	7,00000	542,0000	0,413420	0,140000
389	0,972242	383,0000	43,00000	7,00000	541,0000	0,414502	0,140000
390	0,972186	384,0000	43,00000	7,00000	540,0000	0,415584	0,140000
391	0,972164	385,0000	43,00000	7,00000	539,0000	0,416667	0,140000
392	0,972151	386,0000	43,00000	7,00000	538,0000	0,417749	0,140000
393	0,972098	387,0000	43,00000	7,00000	537,0000	0,418831	0,140000
394	0,972092	388,0000	43,00000	7,00000	536,0000	0,419913	0,140000

395	0,972077	389,0000	43,00000	7,00000	535,0000	0,420996	0,140000
396	0,971958	390,0000	43,00000	7,00000	534,0000	0,422078	0,140000
397	0,971910	391,0000	43,00000	7,00000	533,0000	0,423160	0,140000
398	0,971830	392,0000	43,00000	7,00000	532,0000	0,424242	0,140000
399	0,971789	393,0000	43,00000	7,00000	531,0000	0,425325	0,140000
400	0,971783	394,0000	43,00000	7,00000	530,0000	0,426407	0,140000
401	0,971622	395,0000	43,00000	7,00000	529,0000	0,427489	0,140000
402	0,971436	396,0000	43,00000	7,00000	528,0000	0,428571	0,140000
403	0,971363	397,0000	43,00000	7,00000	527,0000	0,429654	0,140000
404	0,971289	398,0000	43,00000	7,00000	526,0000	0,430736	0,140000
405	0,971269	399,0000	43,00000	7,00000	525,0000	0,431818	0,140000
406	0,971227	400,0000	43,00000	7,00000	524,0000	0,432900	0,140000
407	0,971110	401,0000	43,00000	7,00000	523,0000	0,433983	0,140000
408	0,971102	402,0000	43,00000	7,00000	522,0000	0,435065	0,140000
409	0,971000	403,0000	43,00000	7,00000	521,0000	0,436147	0,140000
410	0,970942	404,0000	43,00000	7,00000	520,0000	0,437229	0,140000
411	0,970936	405,0000	43,00000	7,00000	519,0000	0,438312	0,140000
412	0,970846	406,0000	43,00000	7,00000	518,0000	0,439394	0,140000
413	0,970815	407,0000	43,00000	7,00000	517,0000	0,440476	0,140000
414	0,970783	408,0000	43,00000	7,00000	516,0000	0,441558	0,140000
415	0,970721	409,0000	43,00000	7,00000	515,0000	0,442641	0,140000
416	0,970627	411,0000	43,00000	7,00000	513,0000	0,444805	0,140000
417	0,970574	411,0000	42,00000	8,00000	513,0000	0,444805	0,160000
418	0,970557	412,0000	42,00000	8,00000	512,0000	0,445887	0,160000
419	0,970500	413,0000	42,00000	8,00000	511,0000	0,446970	0,160000
420	0,970491	414,0000	42,00000	8,00000	510,0000	0,448052	0,160000
421	0,970462	415,0000	42,00000	8,00000	509,0000	0,449134	0,160000
422	0,970453	416,0000	42,00000	8,00000	508,0000	0,450216	0,160000
423	0,970358	417,0000	42,00000	8,00000	507,0000	0,451299	0,160000
424	0,970272	418,0000	42,00000	8,00000	506,0000	0,452381	0,160000
425	0,970043	419,0000	42,00000	8,00000	505,0000	0,453463	0,160000
426	0,969986	420,0000	42,00000	8,00000	504,0000	0,454545	0,160000
427	0,969985	421,0000	42,00000	8,00000	503,0000	0,455628	0,160000
428	0,969875	422,0000	42,00000	8,00000	502,0000	0,456710	0,160000
429	0,969782	423,0000	42,00000	8,00000	501,0000	0,457792	0,160000
430	0,969730	424,0000	42,00000	8,00000	500,0000	0,458874	0,160000
431	0,969544	425,0000	42,00000	8,00000	499,0000	0,459957	0,160000
432	0,969457	426,0000	42,00000	8,00000	498,0000	0,461039	0,160000
433	0,969169	427,0000	42,00000	8,00000	497,0000	0,462121	0,160000
434	0,969086	428,0000	42,00000	8,00000	496,0000	0,463203	0,160000
435	0,969037	429,0000	42,00000	8,00000	495,0000	0,464286	0,160000
436	0,968962	430,0000	42,00000	8,00000	494,0000	0,465368	0,160000
437	0,968956	431,0000	42,00000	8,00000	493,0000	0,466450	0,160000
438	0,968948	432,0000	42,00000	8,00000	492,0000	0,467532	0,160000
439	0,968841	433,0000	42,00000	8,00000	491,0000	0,468615	0,160000
440	0,968824	434,0000	42,00000	8,00000	490,0000	0,469697	0,160000

441	0,968793	435,0000	42,00000	8,00000	489,0000	0,470779	0,160000
442	0,968784	436,0000	42,00000	8,00000	488,0000	0,471861	0,160000
443	0,968749	437,0000	42,00000	8,00000	487,0000	0,472944	0,160000
444	0,968614	438,0000	42,00000	8,00000	486,0000	0,474026	0,160000
445	0,968512	439,0000	42,00000	8,00000	485,0000	0,475108	0,160000
446	0,968463	440,0000	42,00000	8,00000	484,0000	0,476190	0,160000
447	0,968454	441,0000	42,00000	8,00000	483,0000	0,477273	0,160000
448	0,968334	442,0000	42,00000	8,00000	482,0000	0,478355	0,160000
449	0,968298	443,0000	42,00000	8,00000	481,0000	0,479437	0,160000
450	0,968286	444,0000	42,00000	8,00000	480,0000	0,480519	0,160000
451	0,968119	445,0000	42,00000	8,00000	479,0000	0,481602	0,160000
452	0,968027	446,0000	42,00000	8,00000	478,0000	0,482684	0,160000
453	0,967966	447,0000	42,00000	8,00000	477,0000	0,483766	0,160000
454	0,967778	448,0000	42,00000	8,00000	476,0000	0,484848	0,160000
455	0,967772	449,0000	42,00000	8,00000	475,0000	0,485931	0,160000
456	0,967713	450,0000	42,00000	8,00000	474,0000	0,487013	0,160000
457	0,967661	451,0000	42,00000	8,00000	473,0000	0,488095	0,160000
458	0,967591	452,0000	42,00000	8,00000	472,0000	0,489177	0,160000
459	0,967509	453,0000	42,00000	8,00000	471,0000	0,490260	0,160000
460	0,967411	454,0000	42,00000	8,00000	470,0000	0,491342	0,160000
461	0,967367	455,0000	42,00000	8,00000	469,0000	0,492424	0,160000
462	0,967331	456,0000	42,00000	8,00000	468,0000	0,493506	0,160000
463	0,967152	457,0000	42,00000	8,00000	467,0000	0,494589	0,160000
464	0,967040	458,0000	42,00000	8,00000	466,0000	0,495671	0,160000
465	0,966907	459,0000	42,00000	8,00000	465,0000	0,496753	0,160000
466	0,966897	460,0000	42,00000	8,00000	464,0000	0,497835	0,160000
467	0,966823	461,0000	42,00000	8,00000	463,0000	0,498918	0,160000
468	0,966794	462,0000	42,00000	8,00000	462,0000	0,500000	0,160000
469	0,966588	463,0000	42,00000	8,00000	461,0000	0,501082	0,160000
470	0,966566	464,0000	42,00000	8,00000	460,0000	0,502165	0,160000
471	0,966327	465,0000	42,00000	8,00000	459,0000	0,503247	0,160000
472	0,966298	466,0000	42,00000	8,00000	458,0000	0,504329	0,160000
473	0,966252	467,0000	42,00000	8,00000	457,0000	0,505411	0,160000
474	0,966218	468,0000	42,00000	8,00000	456,0000	0,506494	0,160000
475	0,965971	469,0000	42,00000	8,00000	455,0000	0,507576	0,160000
476	0,965753	469,0000	41,00000	9,00000	455,0000	0,507576	0,180000
477	0,965536	470,0000	41,00000	9,00000	454,0000	0,508658	0,180000
478	0,965303	471,0000	41,00000	9,00000	453,0000	0,509740	0,180000
479	0,965238	472,0000	41,00000	9,00000	452,0000	0,510823	0,180000
480	0,965146	473,0000	41,00000	9,00000	451,0000	0,511905	0,180000
481	0,965136	474,0000	41,00000	9,00000	450,0000	0,512987	0,180000
482	0,965102	475,0000	41,00000	9,00000	449,0000	0,514069	0,180000
483	0,965037	476,0000	41,00000	9,00000	448,0000	0,515152	0,180000
484	0,965017	477,0000	41,00000	9,00000	447,0000	0,516234	0,180000
485	0,964959	478,0000	41,00000	9,00000	446,0000	0,517316	0,180000
486	0,964944	479,0000	41,00000	9,00000	445,0000	0,518398	0,180000

487	0,964927	480,0000	41,00000	9,00000	444,0000	0,519481	0,180000
488	0,964818	481,0000	41,00000	9,00000	443,0000	0,520563	0,180000
489	0,964769	482,0000	41,00000	9,00000	442,0000	0,521645	0,180000
490	0,964671	483,0000	41,00000	9,00000	441,0000	0,522727	0,180000
491	0,964660	484,0000	41,00000	9,00000	440,0000	0,523810	0,180000
492	0,964601	485,0000	41,00000	9,00000	439,0000	0,524892	0,180000
493	0,964595	486,0000	41,00000	9,00000	438,0000	0,525974	0,180000
494	0,964539	487,0000	41,00000	9,00000	437,0000	0,527056	0,180000
495	0,964534	488,0000	41,00000	9,00000	436,0000	0,528139	0,180000
496	0,964382	489,0000	41,00000	9,00000	435,0000	0,529221	0,180000
497	0,964245	490,0000	41,00000	9,00000	434,0000	0,530303	0,180000
498	0,964228	491,0000	41,00000	9,00000	433,0000	0,531385	0,180000
499	0,964061	492,0000	41,00000	9,00000	432,0000	0,532468	0,180000
500	0,964059	493,0000	41,00000	9,00000	431,0000	0,533550	0,180000
501	0,964015	494,0000	41,00000	9,00000	430,0000	0,534632	0,180000
502	0,963987	495,0000	41,00000	9,00000	429,0000	0,535714	0,180000
503	0,963658	496,0000	41,00000	9,00000	428,0000	0,536797	0,180000
504	0,963550	497,0000	41,00000	9,00000	427,0000	0,537879	0,180000
505	0,963153	498,0000	41,00000	9,00000	426,0000	0,538961	0,180000
506	0,963151	499,0000	41,00000	9,00000	425,0000	0,540043	0,180000
507	0,963130	500,0000	41,00000	9,00000	424,0000	0,541126	0,180000
508	0,963023	501,0000	41,00000	9,00000	423,0000	0,542208	0,180000
509	0,962978	502,0000	41,00000	9,00000	422,0000	0,543290	0,180000
510	0,962886	503,0000	41,00000	9,00000	421,0000	0,544372	0,180000
511	0,962858	504,0000	41,00000	9,00000	420,0000	0,545455	0,180000
512	0,962821	505,0000	41,00000	9,00000	419,0000	0,546537	0,180000
513	0,962811	506,0000	41,00000	9,00000	418,0000	0,547619	0,180000
514	0,962708	507,0000	41,00000	9,00000	417,0000	0,548701	0,180000
515	0,962687	508,0000	41,00000	9,00000	416,0000	0,549784	0,180000
516	0,962684	509,0000	41,00000	9,00000	415,0000	0,550866	0,180000
517	0,962672	510,0000	41,00000	9,00000	414,0000	0,551948	0,180000
518	0,962617	511,0000	41,00000	9,00000	413,0000	0,553030	0,180000
519	0,962558	512,0000	41,00000	9,00000	412,0000	0,554113	0,180000
520	0,962412	513,0000	41,00000	9,00000	411,0000	0,555195	0,180000
521	0,962291	515,0000	41,00000	9,00000	409,0000	0,557359	0,180000
522	0,961881	516,0000	41,00000	9,00000	408,0000	0,558442	0,180000
523	0,961851	517,0000	41,00000	9,00000	407,0000	0,559524	0,180000
524	0,961734	517,0000	40,00000	10,00000	407,0000	0,559524	0,200000
525	0,961693	518,0000	40,00000	10,00000	406,0000	0,560606	0,200000
526	0,961542	519,0000	40,00000	10,00000	405,0000	0,561688	0,200000
527	0,961536	520,0000	40,00000	10,00000	404,0000	0,562771	0,200000
528	0,961416	521,0000	40,00000	10,00000	403,0000	0,563853	0,200000
529	0,961342	522,0000	40,00000	10,00000	402,0000	0,564935	0,200000
530	0,961300	523,0000	40,00000	10,00000	401,0000	0,566017	0,200000
531	0,961253	524,0000	40,00000	10,00000	400,0000	0,567100	0,200000
532	0,961078	525,0000	40,00000	10,00000	399,0000	0,568182	0,200000

533	0,961058	526,0000	40,00000	10,00000	398,0000	0,569264	0,200000
534	0,960960	527,0000	40,00000	10,00000	397,0000	0,570346	0,200000
535	0,960859	528,0000	40,00000	10,00000	396,0000	0,571429	0,200000
536	0,960859	529,0000	40,00000	10,00000	395,0000	0,572511	0,200000
537	0,960614	530,0000	40,00000	10,00000	394,0000	0,573593	0,200000
538	0,960581	531,0000	40,00000	10,00000	393,0000	0,574675	0,200000
539	0,960577	532,0000	40,00000	10,00000	392,0000	0,575758	0,200000
540	0,960576	533,0000	40,00000	10,00000	391,0000	0,576840	0,200000
541	0,960091	534,0000	40,00000	10,00000	390,0000	0,577922	0,200000
542	0,960040	535,0000	40,00000	10,00000	389,0000	0,579004	0,200000
543	0,960013	536,0000	40,00000	10,00000	388,0000	0,580087	0,200000
544	0,959958	537,0000	40,00000	10,00000	387,0000	0,581169	0,200000
545	0,959941	538,0000	40,00000	10,00000	386,0000	0,582251	0,200000
546	0,959884	539,0000	40,00000	10,00000	385,0000	0,583333	0,200000
547	0,959868	540,0000	40,00000	10,00000	384,0000	0,584416	0,200000
548	0,959867	541,0000	40,00000	10,00000	383,0000	0,585498	0,200000
549	0,959780	542,0000	40,00000	10,00000	382,0000	0,586580	0,200000
550	0,959683	543,0000	40,00000	10,00000	381,0000	0,587662	0,200000
551	0,959610	544,0000	40,00000	10,00000	380,0000	0,588745	0,200000
552	0,959556	545,0000	40,00000	10,00000	379,0000	0,589827	0,200000
553	0,959552	546,0000	40,00000	10,00000	378,0000	0,590909	0,200000
554	0,959474	547,0000	40,00000	10,00000	377,0000	0,591991	0,200000
555	0,959274	548,0000	40,00000	10,00000	376,0000	0,593074	0,200000
556	0,959199	549,0000	40,00000	10,00000	375,0000	0,594156	0,200000
557	0,959149	550,0000	40,00000	10,00000	374,0000	0,595238	0,200000
558	0,958882	551,0000	40,00000	10,00000	373,0000	0,596320	0,200000
559	0,958839	552,0000	40,00000	10,00000	372,0000	0,597403	0,200000
560	0,958585	552,0000	39,00000	11,00000	372,0000	0,597403	0,220000
561	0,958567	553,0000	39,00000	11,00000	371,0000	0,598485	0,220000
562	0,958554	554,0000	39,00000	11,00000	370,0000	0,599567	0,220000
563	0,958536	555,0000	39,00000	11,00000	369,0000	0,600649	0,220000
564	0,958516	556,0000	39,00000	11,00000	368,0000	0,601732	0,220000
565	0,958413	557,0000	39,00000	11,00000	367,0000	0,602814	0,220000
566	0,958410	558,0000	39,00000	11,00000	366,0000	0,603896	0,220000
567	0,958296	559,0000	39,00000	11,00000	365,0000	0,604978	0,220000
568	0,958283	560,0000	39,00000	11,00000	364,0000	0,606061	0,220000
569	0,958151	561,0000	39,00000	11,00000	363,0000	0,607143	0,220000
570	0,958089	562,0000	39,00000	11,00000	362,0000	0,608225	0,220000
571	0,957853	563,0000	39,00000	11,00000	361,0000	0,609307	0,220000
572	0,957706	564,0000	39,00000	11,00000	360,0000	0,610390	0,220000
573	0,957548	565,0000	39,00000	11,00000	359,0000	0,611472	0,220000
574	0,957496	566,0000	39,00000	11,00000	358,0000	0,612554	0,220000
575	0,957283	567,0000	39,00000	11,00000	357,0000	0,613636	0,220000
576	0,957275	568,0000	39,00000	11,00000	356,0000	0,614719	0,220000
577	0,957223	569,0000	39,00000	11,00000	355,0000	0,615801	0,220000
578	0,956978	570,0000	39,00000	11,00000	354,0000	0,616883	0,220000

579	0,956678	571,0000	39,00000	11,00000	353,0000	0,617965	0,220000
580	0,956657	572,0000	39,00000	11,00000	352,0000	0,619048	0,220000
581	0,956531	573,0000	39,00000	11,00000	351,0000	0,620130	0,220000
582	0,956526	574,0000	39,00000	11,00000	350,0000	0,621212	0,220000
583	0,956469	574,0000	38,00000	12,00000	350,0000	0,621212	0,240000
584	0,956322	575,0000	38,00000	12,00000	349,0000	0,622294	0,240000
585	0,956288	576,0000	38,00000	12,00000	348,0000	0,623377	0,240000
586	0,956154	577,0000	38,00000	12,00000	347,0000	0,624459	0,240000
587	0,956128	578,0000	38,00000	12,00000	346,0000	0,625541	0,240000
588	0,956121	579,0000	38,00000	12,00000	345,0000	0,626623	0,240000
589	0,956045	580,0000	38,00000	12,00000	344,0000	0,627706	0,240000
590	0,955954	581,0000	38,00000	12,00000	343,0000	0,628788	0,240000
591	0,955875	582,0000	38,00000	12,00000	342,0000	0,629870	0,240000
592	0,955740	583,0000	38,00000	12,00000	341,0000	0,630952	0,240000
593	0,955724	584,0000	38,00000	12,00000	340,0000	0,632035	0,240000
594	0,955365	585,0000	38,00000	12,00000	339,0000	0,633117	0,240000
595	0,955364	586,0000	38,00000	12,00000	338,0000	0,634199	0,240000
596	0,955335	587,0000	38,00000	12,00000	337,0000	0,635281	0,240000
597	0,955318	588,0000	38,00000	12,00000	336,0000	0,636364	0,240000
598	0,955005	589,0000	38,00000	12,00000	335,0000	0,637446	0,240000
599	0,954937	590,0000	38,00000	12,00000	334,0000	0,638528	0,240000
600	0,954838	591,0000	38,00000	12,00000	333,0000	0,639610	0,240000
601	0,954781	592,0000	38,00000	12,00000	332,0000	0,640693	0,240000
602	0,954723	593,0000	38,00000	12,00000	331,0000	0,641775	0,240000
603	0,954710	594,0000	38,00000	12,00000	330,0000	0,642857	0,240000
604	0,954700	595,0000	38,00000	12,00000	329,0000	0,643939	0,240000
605	0,954666	596,0000	38,00000	12,00000	328,0000	0,645022	0,240000
606	0,954661	597,0000	38,00000	12,00000	327,0000	0,646104	0,240000
607	0,954506	598,0000	38,00000	12,00000	326,0000	0,647186	0,240000
608	0,954410	599,0000	38,00000	12,00000	325,0000	0,648268	0,240000
609	0,954078	600,0000	38,00000	12,00000	324,0000	0,649351	0,240000
610	0,953829	601,0000	38,00000	12,00000	323,0000	0,650433	0,240000
611	0,953761	602,0000	38,00000	12,00000	322,0000	0,651515	0,240000
612	0,953580	603,0000	38,00000	12,00000	321,0000	0,652597	0,240000
613	0,953484	604,0000	38,00000	12,00000	320,0000	0,653680	0,240000
614	0,953443	605,0000	38,00000	12,00000	319,0000	0,654762	0,240000
615	0,953432	606,0000	38,00000	12,00000	318,0000	0,655844	0,240000
616	0,953418	607,0000	38,00000	12,00000	317,0000	0,656926	0,240000
617	0,953313	608,0000	38,00000	12,00000	316,0000	0,658009	0,240000
618	0,953263	609,0000	38,00000	12,00000	315,0000	0,659091	0,240000
619	0,953183	610,0000	38,00000	12,00000	314,0000	0,660173	0,240000
620	0,953130	611,0000	38,00000	12,00000	313,0000	0,661255	0,240000
621	0,953123	612,0000	38,00000	12,00000	312,0000	0,662338	0,240000
622	0,953079	613,0000	38,00000	12,00000	311,0000	0,663420	0,240000
623	0,952967	614,0000	38,00000	12,00000	310,0000	0,664502	0,240000
624	0,952650	615,0000	38,00000	12,00000	309,0000	0,665584	0,240000

625	0,952630	616,0000	38,00000	12,00000	308,0000	0,666667	0,240000
626	0,952625	617,0000	38,00000	12,00000	307,0000	0,667749	0,240000
627	0,952521	618,0000	38,00000	12,00000	306,0000	0,668831	0,240000
628	0,952482	619,0000	38,00000	12,00000	305,0000	0,669913	0,240000
629	0,952426	620,0000	38,00000	12,00000	304,0000	0,670996	0,240000
630	0,952289	621,0000	38,00000	12,00000	303,0000	0,672078	0,240000
631	0,952011	622,0000	38,00000	12,00000	302,0000	0,673160	0,240000
632	0,951756	623,0000	38,00000	12,00000	301,0000	0,674242	0,240000
633	0,951618	624,0000	38,00000	12,00000	300,0000	0,675325	0,240000
634	0,951526	625,0000	38,00000	12,00000	299,0000	0,676407	0,240000
635	0,951476	626,0000	38,00000	12,00000	298,0000	0,677489	0,240000
636	0,951470	627,0000	38,00000	12,00000	297,0000	0,678571	0,240000
637	0,951410	628,0000	38,00000	12,00000	296,0000	0,679654	0,240000
638	0,951208	629,0000	38,00000	12,00000	295,0000	0,680736	0,240000
639	0,951200	630,0000	38,00000	12,00000	294,0000	0,681818	0,240000
640	0,951070	631,0000	38,00000	12,00000	293,0000	0,682900	0,240000
641	0,950929	632,0000	38,00000	12,00000	292,0000	0,683983	0,240000
642	0,950837	633,0000	38,00000	12,00000	291,0000	0,685065	0,240000
643	0,950800	634,0000	38,00000	12,00000	290,0000	0,686147	0,240000
644	0,950732	634,0000	37,00000	13,00000	290,0000	0,686147	0,260000
645	0,950729	635,0000	37,00000	13,00000	289,0000	0,687229	0,260000
646	0,950706	636,0000	37,00000	13,00000	288,0000	0,688312	0,260000
647	0,950587	637,0000	37,00000	13,00000	287,0000	0,689394	0,260000
648	0,950548	638,0000	37,00000	13,00000	286,0000	0,690476	0,260000
649	0,950450	639,0000	37,00000	13,00000	285,0000	0,691558	0,260000
650	0,950378	640,0000	37,00000	13,00000	284,0000	0,692641	0,260000
651	0,950324	641,0000	37,00000	13,00000	283,0000	0,693723	0,260000
652	0,950271	641,0000	36,00000	14,00000	283,0000	0,693723	0,280000
653	0,950100	642,0000	36,00000	14,00000	282,0000	0,694805	0,280000
654	0,949921	643,0000	36,00000	14,00000	281,0000	0,695887	0,280000
655	0,949688	644,0000	36,00000	14,00000	280,0000	0,696970	0,280000
656	0,949532	645,0000	36,00000	14,00000	279,0000	0,698052	0,280000
657	0,949381	646,0000	36,00000	14,00000	278,0000	0,699134	0,280000
658	0,949217	647,0000	36,00000	14,00000	277,0000	0,700216	0,280000
659	0,949179	648,0000	36,00000	14,00000	276,0000	0,701299	0,280000
660	0,949020	649,0000	36,00000	14,00000	275,0000	0,702381	0,280000
661	0,949020	650,0000	36,00000	14,00000	274,0000	0,703463	0,280000
662	0,949008	651,0000	36,00000	14,00000	273,0000	0,704545	0,280000
663	0,948972	652,0000	36,00000	14,00000	272,0000	0,705628	0,280000
664	0,948857	653,0000	36,00000	14,00000	271,0000	0,706710	0,280000
665	0,948805	654,0000	36,00000	14,00000	270,0000	0,707792	0,280000
666	0,948802	655,0000	36,00000	14,00000	269,0000	0,708874	0,280000
667	0,948800	656,0000	36,00000	14,00000	268,0000	0,709957	0,280000
668	0,948670	657,0000	36,00000	14,00000	267,0000	0,711039	0,280000
669	0,948596	658,0000	36,00000	14,00000	266,0000	0,712121	0,280000
670	0,948563	659,0000	36,00000	14,00000	265,0000	0,713203	0,280000

671	0,948362	660,0000	36,00000	14,00000	264,0000	0,714286	0,280000
672	0,948250	661,0000	36,00000	14,00000	263,0000	0,715368	0,280000
673	0,948213	662,0000	36,00000	14,00000	262,0000	0,716450	0,280000
674	0,948142	663,0000	36,00000	14,00000	261,0000	0,717532	0,280000
675	0,947970	664,0000	36,00000	14,00000	260,0000	0,718615	0,280000
676	0,947903	665,0000	36,00000	14,00000	259,0000	0,719697	0,280000
677	0,947892	666,0000	36,00000	14,00000	258,0000	0,720779	0,280000
678	0,947769	667,0000	36,00000	14,00000	257,0000	0,721861	0,280000
679	0,947758	668,0000	36,00000	14,00000	256,0000	0,722944	0,280000
680	0,947703	668,0000	35,00000	15,00000	256,0000	0,722944	0,300000
681	0,947698	669,0000	35,00000	15,00000	255,0000	0,724026	0,300000
682	0,947610	670,0000	35,00000	15,00000	254,0000	0,725108	0,300000
683	0,947312	671,0000	35,00000	15,00000	253,0000	0,726190	0,300000
684	0,947034	672,0000	35,00000	15,00000	252,0000	0,727273	0,300000
685	0,946849	673,0000	35,00000	15,00000	251,0000	0,728355	0,300000
686	0,946699	673,0000	34,00000	16,00000	251,0000	0,728355	0,320000
687	0,946605	674,0000	34,00000	16,00000	250,0000	0,729437	0,320000
688	0,946584	675,0000	34,00000	16,00000	249,0000	0,730519	0,320000
689	0,946317	676,0000	34,00000	16,00000	248,0000	0,731602	0,320000
690	0,946237	677,0000	34,00000	16,00000	247,0000	0,732684	0,320000
691	0,946148	678,0000	34,00000	16,00000	246,0000	0,733766	0,320000
692	0,946092	679,0000	34,00000	16,00000	245,0000	0,734848	0,320000
693	0,945981	680,0000	34,00000	16,00000	244,0000	0,735931	0,320000
694	0,945968	681,0000	34,00000	16,00000	243,0000	0,737013	0,320000
695	0,945854	682,0000	34,00000	16,00000	242,0000	0,738095	0,320000
696	0,945562	683,0000	34,00000	16,00000	241,0000	0,739177	0,320000
697	0,945380	684,0000	34,00000	16,00000	240,0000	0,740260	0,320000
698	0,945338	685,0000	34,00000	16,00000	239,0000	0,741342	0,320000
699	0,945278	686,0000	34,00000	16,00000	238,0000	0,742424	0,320000
700	0,945277	687,0000	34,00000	16,00000	237,0000	0,743506	0,320000
701	0,945257	688,0000	34,00000	16,00000	236,0000	0,744589	0,320000
702	0,945208	689,0000	34,00000	16,00000	235,0000	0,745671	0,320000
703	0,945198	690,0000	34,00000	16,00000	234,0000	0,746753	0,320000
704	0,945135	691,0000	34,00000	16,00000	233,0000	0,747835	0,320000
705	0,945035	692,0000	34,00000	16,00000	232,0000	0,748918	0,320000
706	0,944789	693,0000	34,00000	16,00000	231,0000	0,750000	0,320000
707	0,944670	693,0000	33,00000	17,00000	231,0000	0,750000	0,340000
708	0,944635	694,0000	33,00000	17,00000	230,0000	0,751082	0,340000
709	0,944615	695,0000	33,00000	17,00000	229,0000	0,752165	0,340000
710	0,944340	696,0000	33,00000	17,00000	228,0000	0,753247	0,340000
711	0,944168	697,0000	33,00000	17,00000	227,0000	0,754329	0,340000
712	0,943725	698,0000	33,00000	17,00000	226,0000	0,755411	0,340000
713	0,943563	699,0000	33,00000	17,00000	225,0000	0,756494	0,340000
714	0,943556	699,0000	32,00000	18,00000	225,0000	0,756494	0,360000
715	0,943430	699,0000	31,00000	19,00000	225,0000	0,756494	0,380000
716	0,943383	700,0000	31,00000	19,00000	224,0000	0,757576	0,380000

717	0,943349	701,0000	31,00000	19,00000	223,0000	0,758658	0,380000
718	0,943303	702,0000	31,00000	19,00000	222,0000	0,759740	0,380000
719	0,942549	703,0000	31,00000	19,00000	221,0000	0,760823	0,380000
720	0,942474	704,0000	31,00000	19,00000	220,0000	0,761905	0,380000
721	0,942198	705,0000	31,00000	19,00000	219,0000	0,762987	0,380000
722	0,942117	705,0000	30,00000	20,00000	219,0000	0,762987	0,400000
723	0,942026	706,0000	30,00000	20,00000	218,0000	0,764069	0,400000
724	0,941770	707,0000	30,00000	20,00000	217,0000	0,765152	0,400000
725	0,941761	708,0000	30,00000	20,00000	216,0000	0,766234	0,400000
726	0,940928	709,0000	30,00000	20,00000	215,0000	0,767316	0,400000
727	0,940776	710,0000	30,00000	20,00000	214,0000	0,768398	0,400000
728	0,940739	711,0000	30,00000	20,00000	213,0000	0,769481	0,400000
729	0,940640	711,0000	29,00000	21,00000	213,0000	0,769481	0,420000
730	0,940602	712,0000	29,00000	21,00000	212,0000	0,770563	0,420000
731	0,940600	712,0000	28,00000	22,00000	212,0000	0,770563	0,440000
732	0,940196	713,0000	28,00000	22,00000	211,0000	0,771645	0,440000
733	0,939984	714,0000	28,00000	22,00000	210,0000	0,772727	0,440000
734	0,939869	715,0000	28,00000	22,00000	209,0000	0,773810	0,440000
735	0,939648	716,0000	28,00000	22,00000	208,0000	0,774892	0,440000
736	0,939070	717,0000	28,00000	22,00000	207,0000	0,775974	0,440000
737	0,939004	718,0000	28,00000	22,00000	206,0000	0,777056	0,440000
738	0,938712	719,0000	28,00000	22,00000	205,0000	0,778139	0,440000
739	0,937998	720,0000	28,00000	22,00000	204,0000	0,779221	0,440000
740	0,937440	721,0000	28,00000	22,00000	203,0000	0,780303	0,440000
741	0,937386	722,0000	28,00000	22,00000	202,0000	0,781385	0,440000
742	0,937212	723,0000	28,00000	22,00000	201,0000	0,782468	0,440000
743	0,937110	724,0000	28,00000	22,00000	200,0000	0,783550	0,440000
744	0,936996	725,0000	28,00000	22,00000	199,0000	0,784632	0,440000
745	0,936910	726,0000	28,00000	22,00000	198,0000	0,785714	0,440000
746	0,936286	727,0000	28,00000	22,00000	197,0000	0,786797	0,440000
747	0,936202	728,0000	28,00000	22,00000	196,0000	0,787879	0,440000
748	0,935846	729,0000	28,00000	22,00000	195,0000	0,788961	0,440000
749	0,935471	730,0000	28,00000	22,00000	194,0000	0,790043	0,440000
750	0,935252	731,0000	28,00000	22,00000	193,0000	0,791126	0,440000
751	0,935104	732,0000	28,00000	22,00000	192,0000	0,792208	0,440000
752	0,934493	733,0000	28,00000	22,00000	191,0000	0,793290	0,440000
753	0,934306	734,0000	28,00000	22,00000	190,0000	0,794372	0,440000
754	0,934196	735,0000	28,00000	22,00000	189,0000	0,795455	0,440000
755	0,933975	736,0000	28,00000	22,00000	188,0000	0,796537	0,440000
756	0,933380	737,0000	28,00000	22,00000	187,0000	0,797619	0,440000
757	0,932352	738,0000	28,00000	22,00000	186,0000	0,798701	0,440000
758	0,932075	739,0000	28,00000	22,00000	185,0000	0,799784	0,440000
759	0,931996	739,0000	27,00000	23,00000	185,0000	0,799784	0,460000
760	0,931819	740,0000	27,00000	23,00000	184,0000	0,800866	0,460000
761	0,931798	741,0000	27,00000	23,00000	183,0000	0,801948	0,460000
762	0,931217	742,0000	27,00000	23,00000	182,0000	0,803030	0,460000

763	0,931026	743,0000	27,00000	23,00000	181,0000	0,804113	0,460000
764	0,930859	744,0000	27,00000	23,00000	180,0000	0,805195	0,460000
765	0,930720	745,0000	27,00000	23,00000	179,0000	0,806277	0,460000
766	0,930626	746,0000	27,00000	23,00000	178,0000	0,807359	0,460000
767	0,930224	747,0000	27,00000	23,00000	177,0000	0,808442	0,460000
768	0,930121	748,0000	27,00000	23,00000	176,0000	0,809524	0,460000
769	0,930077	749,0000	27,00000	23,00000	175,0000	0,810606	0,460000
770	0,930014	750,0000	27,00000	23,00000	174,0000	0,811688	0,460000
771	0,929915	751,0000	27,00000	23,00000	173,0000	0,812771	0,460000
772	0,929871	752,0000	27,00000	23,00000	172,0000	0,813853	0,460000
773	0,929315	753,0000	27,00000	23,00000	171,0000	0,814935	0,460000
774	0,929125	754,0000	27,00000	23,00000	170,0000	0,816017	0,460000
775	0,928476	755,0000	27,00000	23,00000	169,0000	0,817100	0,460000
776	0,928403	756,0000	27,00000	23,00000	168,0000	0,818182	0,460000
777	0,928181	757,0000	27,00000	23,00000	167,0000	0,819264	0,460000
778	0,928109	758,0000	27,00000	23,00000	166,0000	0,820346	0,460000
779	0,928038	759,0000	27,00000	23,00000	165,0000	0,821429	0,460000
780	0,926839	760,0000	27,00000	23,00000	164,0000	0,822511	0,460000
781	0,926794	761,0000	27,00000	23,00000	163,0000	0,823593	0,460000
782	0,925915	762,0000	27,00000	23,00000	162,0000	0,824675	0,460000
783	0,925867	763,0000	27,00000	23,00000	161,0000	0,825758	0,460000
784	0,925723	764,0000	27,00000	23,00000	160,0000	0,826840	0,460000
785	0,925482	765,0000	27,00000	23,00000	159,0000	0,827922	0,460000
786	0,925376	766,0000	27,00000	23,00000	158,0000	0,829004	0,460000
787	0,925194	767,0000	27,00000	23,00000	157,0000	0,830087	0,460000
788	0,924961	768,0000	27,00000	23,00000	156,0000	0,831169	0,460000
789	0,924953	769,0000	27,00000	23,00000	155,0000	0,832251	0,460000
790	0,924858	770,0000	27,00000	23,00000	154,0000	0,833333	0,460000
791	0,924813	771,0000	27,00000	23,00000	153,0000	0,834416	0,460000
792	0,924639	772,0000	27,00000	23,00000	152,0000	0,835498	0,460000
793	0,924559	773,0000	27,00000	23,00000	151,0000	0,836580	0,460000
794	0,924351	774,0000	27,00000	23,00000	150,0000	0,837662	0,460000
795	0,924145	775,0000	27,00000	23,00000	149,0000	0,838745	0,460000
796	0,923866	776,0000	27,00000	23,00000	148,0000	0,839827	0,460000
797	0,923349	776,0000	26,00000	24,00000	148,0000	0,839827	0,480000
798	0,923318	777,0000	26,00000	24,00000	147,0000	0,840909	0,480000
799	0,922878	778,0000	26,00000	24,00000	146,0000	0,841991	0,480000
800	0,922851	779,0000	26,00000	24,00000	145,0000	0,843074	0,480000
801	0,922716	780,0000	26,00000	24,00000	144,0000	0,844156	0,480000
802	0,922350	781,0000	26,00000	24,00000	143,0000	0,845238	0,480000
803	0,920405	782,0000	26,00000	24,00000	142,0000	0,846320	0,480000
804	0,920170	783,0000	26,00000	24,00000	141,0000	0,847403	0,480000
805	0,919920	784,0000	26,00000	24,00000	140,0000	0,848485	0,480000
806	0,919632	785,0000	26,00000	24,00000	139,0000	0,849567	0,480000
807	0,919519	786,0000	26,00000	24,00000	138,0000	0,850649	0,480000
808	0,918975	787,0000	26,00000	24,00000	137,0000	0,851732	0,480000

809	0,918454	788,0000	26,00000	24,00000	136,0000	0,852814	0,480000
810	0,917495	789,0000	26,00000	24,00000	135,0000	0,853896	0,480000
811	0,917403	789,0000	25,00000	25,00000	135,0000	0,853896	0,500000
812	0,917194	790,0000	25,00000	25,00000	134,0000	0,854978	0,500000
813	0,916383	791,0000	25,00000	25,00000	133,0000	0,856061	0,500000
814	0,916303	792,0000	25,00000	25,00000	132,0000	0,857143	0,500000
815	0,915577	793,0000	25,00000	25,00000	131,0000	0,858225	0,500000
816	0,914553	794,0000	25,00000	25,00000	130,0000	0,859307	0,500000
817	0,914467	794,0000	24,00000	26,00000	130,0000	0,859307	0,520000
818	0,913157	795,0000	24,00000	26,00000	129,0000	0,860390	0,520000
819	0,912754	796,0000	24,00000	26,00000	128,0000	0,861472	0,520000
820	0,912223	797,0000	24,00000	26,00000	127,0000	0,862554	0,520000
821	0,911969	797,0000	23,00000	27,00000	127,0000	0,862554	0,540000
822	0,911833	798,0000	23,00000	27,00000	126,0000	0,863636	0,540000
823	0,911153	799,0000	23,00000	27,00000	125,0000	0,864719	0,540000
824	0,910826	800,0000	23,00000	27,00000	124,0000	0,865801	0,540000
825	0,910021	801,0000	23,00000	27,00000	123,0000	0,866883	0,540000
826	0,909861	802,0000	23,00000	27,00000	122,0000	0,867965	0,540000
827	0,909583	802,0000	22,00000	28,00000	122,0000	0,867965	0,560000
828	0,909264	803,0000	22,00000	28,00000	121,0000	0,869048	0,560000
829	0,909065	804,0000	22,00000	28,00000	120,0000	0,870130	0,560000
830	0,908484	805,0000	22,00000	28,00000	119,0000	0,871212	0,560000
831	0,908187	806,0000	22,00000	28,00000	118,0000	0,872294	0,560000
832	0,908117	807,0000	22,00000	28,00000	117,0000	0,873377	0,560000
833	0,907915	808,0000	22,00000	28,00000	116,0000	0,874459	0,560000
834	0,907433	809,0000	22,00000	28,00000	115,0000	0,875541	0,560000
835	0,907011	810,0000	22,00000	28,00000	114,0000	0,876623	0,560000
836	0,906198	811,0000	22,00000	28,00000	113,0000	0,877706	0,560000
837	0,905290	812,0000	22,00000	28,00000	112,0000	0,878788	0,560000
838	0,904595	813,0000	22,00000	28,00000	111,0000	0,879870	0,560000
839	0,904102	814,0000	22,00000	28,00000	110,0000	0,880952	0,560000
840	0,904085	815,0000	22,00000	28,00000	109,0000	0,882035	0,560000
841	0,903492	816,0000	22,00000	28,00000	108,0000	0,883117	0,560000
842	0,902874	817,0000	22,00000	28,00000	107,0000	0,884199	0,560000
843	0,901842	818,0000	22,00000	28,00000	106,0000	0,885281	0,560000
844	0,901484	819,0000	22,00000	28,00000	105,0000	0,886364	0,560000
845	0,901208	820,0000	22,00000	28,00000	104,0000	0,887446	0,560000
846	0,901044	821,0000	22,00000	28,00000	103,0000	0,888528	0,560000
847	0,900676	822,0000	22,00000	28,00000	102,0000	0,889610	0,560000
848	0,899452	823,0000	22,00000	28,00000	101,0000	0,890693	0,560000
849	0,899250	824,0000	22,00000	28,00000	100,0000	0,891775	0,560000
850	0,898828	825,0000	22,00000	28,00000	99,0000	0,892857	0,560000
851	0,898788	826,0000	22,00000	28,00000	98,0000	0,893939	0,560000
852	0,898562	827,0000	22,00000	28,00000	97,0000	0,895022	0,560000
853	0,898281	828,0000	22,00000	28,00000	96,0000	0,896104	0,560000
854	0,898194	829,0000	22,00000	28,00000	95,0000	0,897186	0,560000

855	0,898194	829,0000	21,00000	29,00000	95,0000	0,897186	0,580000
856	0,897442	830,0000	21,00000	29,00000	94,0000	0,898268	0,580000
857	0,896715	831,0000	21,00000	29,00000	93,0000	0,899351	0,580000
858	0,896643	832,0000	21,00000	29,00000	92,0000	0,900433	0,580000
859	0,896361	833,0000	21,00000	29,00000	91,0000	0,901515	0,580000
860	0,894870	834,0000	21,00000	29,00000	90,0000	0,902597	0,580000
861	0,894274	835,0000	21,00000	29,00000	89,0000	0,903680	0,580000
862	0,894011	836,0000	21,00000	29,00000	88,0000	0,904762	0,580000
863	0,893940	837,0000	21,00000	29,00000	87,0000	0,905844	0,580000
864	0,893294	838,0000	21,00000	29,00000	86,0000	0,906926	0,580000
865	0,893217	839,0000	21,00000	29,00000	85,0000	0,908009	0,580000
866	0,892548	840,0000	21,00000	29,00000	84,0000	0,909091	0,580000
867	0,891698	841,0000	21,00000	29,00000	83,0000	0,910173	0,580000
868	0,890860	842,0000	21,00000	29,00000	82,0000	0,911255	0,580000
869	0,890691	843,0000	21,00000	29,00000	81,0000	0,912338	0,580000
870	0,890659	844,0000	21,00000	29,00000	80,0000	0,913420	0,580000
871	0,890529	845,0000	21,00000	29,00000	79,0000	0,914502	0,580000
872	0,889728	846,0000	21,00000	29,00000	78,0000	0,915584	0,580000
873	0,889477	847,0000	21,00000	29,00000	77,0000	0,916667	0,580000
874	0,889441	848,0000	21,00000	29,00000	76,0000	0,917749	0,580000
875	0,889357	849,0000	21,00000	29,00000	75,0000	0,918831	0,580000
876	0,889270	850,0000	21,00000	29,00000	74,0000	0,919913	0,580000
877	0,888618	850,0000	20,00000	30,00000	74,0000	0,919913	0,600000
878	0,888283	851,0000	20,00000	30,00000	73,0000	0,920996	0,600000
879	0,886997	852,0000	20,00000	30,00000	72,0000	0,922078	0,600000
880	0,886897	852,0000	19,00000	31,00000	72,0000	0,922078	0,620000
881	0,885560	853,0000	19,00000	31,00000	71,0000	0,923160	0,620000
882	0,885029	854,0000	19,00000	31,00000	70,0000	0,924242	0,620000
883	0,884179	855,0000	19,00000	31,00000	69,0000	0,925325	0,620000
884	0,882584	856,0000	19,00000	31,00000	68,0000	0,926407	0,620000
885	0,881534	857,0000	19,00000	31,00000	67,0000	0,927489	0,620000
886	0,879994	858,0000	19,00000	31,00000	66,0000	0,928571	0,620000
887	0,879909	858,0000	18,00000	32,00000	66,0000	0,928571	0,640000
888	0,878492	859,0000	18,00000	32,00000	65,0000	0,929654	0,640000
889	0,878136	860,0000	18,00000	32,00000	64,0000	0,930736	0,640000
890	0,878023	861,0000	18,00000	32,00000	63,0000	0,931818	0,640000
891	0,877695	862,0000	18,00000	32,00000	62,0000	0,932900	0,640000
892	0,877542	863,0000	18,00000	32,00000	61,0000	0,933983	0,640000
893	0,876358	864,0000	18,00000	32,00000	60,0000	0,935065	0,640000
894	0,874717	865,0000	18,00000	32,00000	59,0000	0,936147	0,640000
895	0,874647	866,0000	18,00000	32,00000	58,0000	0,937229	0,640000
896	0,873663	867,0000	18,00000	32,00000	57,0000	0,938312	0,640000
897	0,872456	868,0000	18,00000	32,00000	56,0000	0,939394	0,640000
898	0,871720	868,0000	17,00000	33,00000	56,0000	0,939394	0,660000
899	0,867239	869,0000	17,00000	33,00000	55,0000	0,940476	0,660000
900	0,866678	870,0000	17,00000	33,00000	54,0000	0,941558	0,660000

901	0,864309	871,0000	17,00000	33,00000	53,0000	0,942641	0,660000
902	0,863494	872,0000	17,00000	33,00000	52,0000	0,943723	0,660000
903	0,862633	873,0000	17,00000	33,00000	51,0000	0,944805	0,660000
904	0,861389	874,0000	17,00000	33,00000	50,0000	0,945887	0,660000
905	0,861287	875,0000	17,00000	33,00000	49,0000	0,946970	0,660000
906	0,860611	876,0000	17,00000	33,00000	48,0000	0,948052	0,660000
907	0,860297	877,0000	17,00000	33,00000	47,0000	0,949134	0,660000
908	0,859687	878,0000	17,00000	33,00000	46,0000	0,950216	0,660000
909	0,858115	878,0000	16,00000	34,00000	46,0000	0,950216	0,680000
910	0,857707	878,0000	15,00000	35,00000	46,0000	0,950216	0,700000
911	0,857556	879,0000	15,00000	35,00000	45,0000	0,951299	0,700000
912	0,857256	880,0000	15,00000	35,00000	44,0000	0,952381	0,700000
913	0,856970	881,0000	15,00000	35,00000	43,0000	0,953463	0,700000
914	0,856596	882,0000	15,00000	35,00000	42,0000	0,954545	0,700000
915	0,855392	883,0000	15,00000	35,00000	41,0000	0,955628	0,700000
916	0,854830	883,0000	14,00000	36,00000	41,0000	0,955628	0,720000
917	0,853777	884,0000	14,00000	36,00000	40,0000	0,956710	0,720000
918	0,853605	885,0000	14,00000	36,00000	39,0000	0,957792	0,720000
919	0,851031	885,0000	13,00000	37,00000	39,0000	0,957792	0,740000
920	0,850705	886,0000	13,00000	37,00000	38,0000	0,958874	0,740000
921	0,850702	887,0000	13,00000	37,00000	37,0000	0,959957	0,740000
922	0,848033	888,0000	13,00000	37,00000	36,0000	0,961039	0,740000
923	0,840939	889,0000	13,00000	37,00000	35,0000	0,962121	0,740000
924	0,839767	889,0000	12,00000	38,00000	35,0000	0,962121	0,760000
925	0,835255	889,0000	11,00000	39,00000	35,0000	0,962121	0,780000
926	0,834782	890,0000	11,00000	39,00000	34,0000	0,963203	0,780000
927	0,834765	891,0000	11,00000	39,00000	33,0000	0,964286	0,780000
928	0,834733	892,0000	11,00000	39,00000	32,0000	0,965368	0,780000
929	0,834376	892,0000	10,00000	40,00000	32,0000	0,965368	0,800000
930	0,834174	893,0000	10,00000	40,00000	31,0000	0,966450	0,800000
931	0,833769	894,0000	10,00000	40,00000	30,0000	0,967532	0,800000
932	0,833331	895,0000	10,00000	40,00000	29,0000	0,968615	0,800000
933	0,826800	896,0000	10,00000	40,00000	28,0000	0,969697	0,800000
934	0,825356	897,0000	10,00000	40,00000	27,0000	0,970779	0,800000
935	0,820529	898,0000	9,00000	41,00000	26,0000	0,971861	0,820000
936	0,815036	899,0000	9,00000	41,00000	25,0000	0,972944	0,820000
937	0,814772	900,0000	9,00000	41,00000	24,0000	0,974026	0,820000
938	0,814241	901,0000	9,00000	41,00000	23,0000	0,975108	0,820000
939	0,811248	902,0000	9,00000	41,00000	22,0000	0,976190	0,820000
940	0,804535	903,0000	9,00000	41,00000	21,0000	0,977273	0,820000
941	0,798903	904,0000	9,00000	41,00000	20,0000	0,978355	0,820000
942	0,797326	904,0000	8,00000	42,00000	20,0000	0,978355	0,840000
943	0,796314	905,0000	8,00000	42,00000	19,0000	0,979437	0,840000
944	0,795492	906,0000	8,00000	42,00000	18,0000	0,980519	0,840000
945	0,794721	907,0000	8,00000	42,00000	17,0000	0,981602	0,840000
946	0,793689	908,0000	8,00000	42,00000	16,0000	0,982684	0,840000

947	0,792601	909,0000	8,00000	42,00000	15,0000	0,983766	0,840000
948	0,791981	910,0000	8,00000	42,00000	14,0000	0,984848	0,840000
949	0,791925	910,0000	7,00000	43,00000	14,0000	0,984848	0,860000
950	0,790046	911,0000	7,00000	43,00000	13,0000	0,985931	0,860000
951	0,789414	912,0000	7,00000	43,00000	12,0000	0,987013	0,860000
952	0,788735	912,0000	6,00000	44,00000	12,0000	0,987013	0,880000
953	0,786431	913,0000	6,00000	44,00000	11,0000	0,988095	0,880000
954	0,785152	914,0000	6,00000	44,00000	10,0000	0,989177	0,880000
955	0,781791	915,0000	6,00000	44,00000	9,0000	0,990260	0,880000
956	0,779792	915,0000	5,00000	45,00000	9,0000	0,990260	0,900000
957	0,778193	916,0000	5,00000	45,00000	8,0000	0,991342	0,900000
958	0,775831	916,0000	4,00000	46,00000	8,0000	0,991342	0,920000
959	0,774601	917,0000	4,00000	46,00000	7,0000	0,992424	0,920000
960	0,771095	918,0000	4,00000	46,00000	6,0000	0,993506	0,920000
961	0,769812	919,0000	4,00000	46,00000	5,0000	0,994589	0,920000
962	0,769099	919,0000	3,00000	47,00000	5,0000	0,994589	0,940000
963	0,748990	919,0000	2,00000	48,00000	5,0000	0,994589	0,960000
964	0,734809	920,0000	2,00000	48,00000	4,0000	0,995671	0,960000
965	0,711434	921,0000	2,00000	48,00000	3,0000	0,996753	0,960000
966	0,705303	921,0000	1,00000	49,00000	3,0000	0,996753	0,980000
967	0,645052	922,0000	1,00000	49,00000	2,0000	0,997835	0,980000
968	0,633597	923,0000	1,00000	49,00000	2,0000	0,998918	0,980000
969	0,588987	923,0000	0,00000	49,00000	2,0000	0,998918	0,980000
970	0,125689	923,0000	0,00000	49,00000	2,0000	0,999254	0,990000
971	0,723598	923,0000	0,00000	50,00000	1,0000	0,978944	0,990000
972	0,824303	924,0000	0,00000	50,00000	1,0000	0,997721	0,990000
973	0,892593	924,0000	0,00000	50,00000	1,0000	0,954123	0,990000
973	0,705221	924,0000	0,00000	50,00000	1,0000	0,996524	0,990000
974	0,745603	924,0000	0,00000	50,00000	1,0000	0,998754	1,000000
975	0,636697	924,0000	1,00000	49,00000	1,0000	0,998918	1,000000
976	0,589256	924,0000	0,00000	50,00000	1,0000	0,998918	1,000000
977	0,134689	924,0000	0,00000	50,00000	0,0000	1,000000	1,000000
978	0,236548	924,0000	0,00000	50,00000	0,0000	1,000000	1,000000
979	0,543289	924,0000	0,00000	50,00000	0,0000	1,000000	1,000000

No – number of cows
BHB0/1 - BHB – 1 sick cows; 0 healthy cows
ROC - Receiver Operating Characteristic
True negative rate – specificity
True positive rate – sensitivity
Cutoff point – defined as a sensitivity + specificity - 1
